# Phylogeny-guided genome mining of roseocin family lantibiotics to generate improved variants of roseocin

**DOI:** 10.1186/s13568-023-01536-9

**Published:** 2023-03-20

**Authors:** Sandeep Chaudhary, Shweta Kishen, Mangal Singh, Sunanda Jassal, Reeva Pathania, Kalpana Bisht, Dipti Sareen

**Affiliations:** 1 Department of Biochemistry, Panjab University, Chandigarh, 160014 India; 2 Department of Biosciences and Bioengineering, Indian Institute of Technology Roorkee, Roorkee, 247667 India

**Keywords:** *Actinobacteria*, Lantibiotics, Phylum, Post-translational modification, Variants

## Abstract

Roseocin, the two-peptide lantibiotic from *Streptomyces roseosporus*, carries extensive intramolecular (methyl)lanthionine bridging in the peptides and exhibits synergistic antibacterial activity against clinically relevant Gram-positive pathogens. Both peptides have a conserved leader but a diverse core region. The biosynthesis of roseocin involves post-translational modification of the two precursor peptides by a single promiscuous lanthipeptide synthetase, RosM, to install an indispensable disulfide bond in the Rosα core along with four and six thioether rings in Rosα and Rosβ cores, respectively. RosM homologs in the phylum *actinobacteria* were identified here to reveal twelve other members of the roseocin family which diverged into three types of biosynthetic gene clusters (BGCs). Further, the evolutionary rate among the BGC variants and analysis of variability within the core peptide versus leader peptide revealed a phylum-dependent lanthipeptide evolution. Analysis of horizontal gene transfer revealed its role in the generation of core peptide diversity. The naturally occurring diverse congeners of roseocin peptides identified from the mined novel BGCs were carefully aligned to identify the conserved sites and the substitutions in the core peptide region. These selected sites in the Rosα peptide were mutated for permitted substitutions, expressed heterologously in *E. coli*, and post-translationally modified by RosM in vivo. Despite a limited number of generated variants, two variants, RosαL8F and RosαL8W exhibited significantly improved inhibitory activity in a species-dependent manner compared to the wild-type roseocin. Our study proves that a natural repository of evolved variants of roseocin is present in nature and the key variations can be used to generate improved variants.

**Supplementary Information:**

The online version contains supplementary material available at 10.1186/s13568-023-01536-9.

## Keypoints

- Taxonomically distant species have a wide diversity of roseocin family lantibiotics.
- The phylogeny-guided approach opens new possibilities for RiPPs bioengineering.
- Bioactive variants of roseocin were generated using semi-in vitro reconstitution.

**Supplementary Information:**

The online version contains supplementary material available at 10.1186/s13568-023-01536-9.

## Introduction

Nature has bestowed upon the bacterial species an arsenal of antimicrobial moieties to benefit the host in intraspecies or interspecies competition under the limited natural resources of an ecological niche. The human race has benefited profoundly from these microbial conflicts with the discovery of novel antimicrobials to combat bacterial infections. However, extensive and indiscriminate use of antimicrobials has fuelled the rapid spread of antimicrobial resistance (AMR), rendering antimicrobials of similar type ineffective. One of the ways to solve this crisis is to discover other alternative classes of antimicrobials having high efficacy against these multi-drug-resistant (MDR) pathogens along with a scope for possible bioengineering in the future. Lantibiotics (or antimicrobial lanthipeptides) are one such class that belong to ribosomally synthesized and post-translationally modified peptides (RiPPs), and their heterologous expression in *E. coli* based platforms has facilitated the formation of new-to-nature molecules by site-directed/random mutagenesis and combinatorial biosynthesis (Iacovelli et al. 2022).

**Fig. 1:**
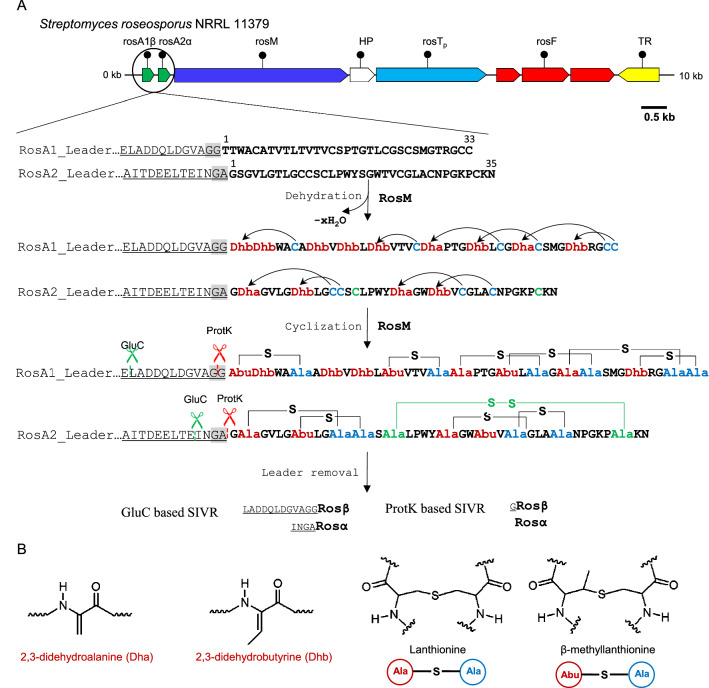
Biosynthesis of the two-component lantibiotic roseocin. **A** Roseocin is constituted of two precursor peptides, RosA1β and RosA2α, which are post-translationally modified by RosM in two steps (dehydration followed by cyclization). This is followed by leader removal in vitro using commercially available proteases like endoproteinase GluC or proteinase K (Singh et al. 2020). The ring topology of roseocin peptides is supported by mass spectrometry and bioinformatic analysis as per the current study. **B** Common PTMs in the biosynthesis of lanthipeptide involves the dehydration of serine and threonine amino acid residues to 2,3-dehydroalanine (Dha) and 2,3-dehydrobutyrine (Dhb), respectively. Michael-type addition of Cys to Dha leads to lanthionine (Lan), while addition to Dhb results in a methyllanthionine (MeLan) ring. *Abu* aminobutyrate, *Ala* Alanine, *SIVR* semi-in vitro reconstitution, *HP* Hypothetical Protein, *RosTp* dual function peptidase-domain containing transporter, *RosF* group of immunity proteins (in red colour), *TR* Transcriptional Regulator

Lanthipeptides are decorated with characteristic thioether rings that render them proteolytically stable and target-accessible (Montalbán-López et al. 2021). The synthesis of a lanthipeptide begins from the precursor peptide (LanA), a genetically encoded linear peptide (Fig. 1A), which is a variable component in the biosynthetic gene clusters (BGCs), along with the lanthipeptide synthetase, immunity proteins, and other tailoring enzymes. The precursor peptide has an N-terminal leader peptide and a C-terminal core, separated by a proteolytic site for removal of the leader peptide following the complete post-translational modifications (PTMs) of the core peptide (McAuliffe et al. 2001; Oman and van der Donk 2010). A lanthipeptide leader region is essential in guiding the core peptide at the active site of the lanthipeptide synthetase for ring installation (Lubelski et al. 2009). The lanthionine rings are installed in two steps; firstly, the dehydration of Ser/Thr residues occurs, which is followed by intra-peptide Michael addition of cysteine residues (cyclization) to form lanthionine/methyllanthionine rings (Fig. 1B) (Xie et al. 2004). Based on the structure and function of lanthipeptide synthetase, four lanthipeptide classes (class I-IV) have been defined (Arnison et al. 2013; Repka et al. 2017). To process class I lanthipeptides, two separate dehydration (LanB) and cyclization (LanC) enzymes are encoded in the BGC. For class II lanthipeptides, a single bifunctional domain-containing enzyme, LanM is present with an N-terminal dehydratase and a C-terminal cyclase domain. For class III and IV lanthipeptides, a single enzyme (LanKC and LanL, respectively) with trifunctional domains, i.e. lyase, kinase, and cyclase is required. A detailed description of the mechanism and structure of the lanthipeptide synthetases is provided by Repka et al. 2017.

The LanMs are of particular interest not only because of their bifunctional domain structure but also their substrate promiscuity. Evolutionary studies have shown a significant divergence among LanMs based on the natural substrate promiscuity, defined by the number of precursor peptides encoded within a single genome (Zhang et al. 2012). Highly promiscuous LanMs possess a CCG motif, instead of the CHG motif, for binding essential Zn^2+^ ion at the active site (Zhang et al. 2014). ProcM is the first example of a CCG motif LanM with an ability to process 29 diverse precursor peptides, genetically encoded and spread throughout the genome of *Prochlorococcus* MIT9313 (Li et al. 2010). In an exciting study by Yang et al. 2018, the substrate tolerance and catalytic fidelity of ProcM were utilized to generate a library of 1.07 × 10^9^ non-native variants. The 36 peptides examined out of this library (made from ProcA2.8, which is one of the 30 naturally encoded substrates of ProcM) were all found to have a faithful installation of the two-ring scaffold, thus establishing the possibility of generating lanthipeptide libraries with a wide range of chemical diversity by utilizing such promiscuous LanMs. Another recently reported CCG motif containing LanM, SyncM processes 79 naturally encoded precursor peptides and is the most promiscuous lanthipeptide synthetase known so far (Arias-Orozco et al. 2021).

Unlike CCG motif LanMs, comparatively limited promiscuity is exhibited by CHG motif containing LanMs, which are involved in the biosynthesis of two-component lantibiotics. These lantibiotics bifurcate the action of a single-component lantibiotic into alpha- and beta-peptides in their synergistic antimicrobial mode of action (Oman and Van Der Donk 2009; Bakhtiary et al. 2017). The alpha-peptide is responsible for anchoring to lipid II in the outer leaflet of the cytoplasmic membrane, and the beta-peptide potentiates the alpha:lipid II complex to form a heteromeric complex leading to the rapid cell lysis (Bakhtiary et al. 2017; Oman et al. 2011). Roseocin is a recent example of a two-component lantibiotic that results from the post-translational modification of two precursor peptides by a single promiscuous CHG-type RosM (Singh et al. 2020). Such promiscuity is rare among other two-component lantibiotics, as all the lacticin 3147 family lantibiotics have two separate dedicated CHG type LanMs for the two precursors (Mcclerren et al. 2006; Xin et al. 2016). The only characterized examples of single CHG-type LanM and two precursors are cytolysin (Coburn and Gilmore 2003), carnolysin (Lohans et al. 2014) and bicereucin (Huo and Van Der Donk 2016), but all of these belong to the phylum *firmicutes*. However roseocin, identified from *S. roseosporus* NRRL 11379 is the sole example, from the phylum *actinobacteria*, of single LanM and two precursors that lack homology to any of the known two-component lantibiotics (Singh et al. 2020). Owing to the antimicrobial potential of roseocin against MDR strains and having previously developed its heterologous expression system in *E. coli*, we wanted to explore its variants with improved antibacterial activity. Instead of random mutagenesis, we utilized the sequence diversity among the identified roseocin homologs. The study of phylogenetically related BGCs is a promising way to obtain an extensive collection of divergent congeners resulting from an evolutionary pressure (Wang et al. 2022a). In a quest to identify homologs of roseocin, we devised a methodology using lanthipeptide synthetases, which have evolved in a phylum-dependent manner (Zhang et al. 2012; Yu et al. 2013). An earlier study by Walker et al. 2020 utilized the LanC domain of lanthipeptide synthetase as a query and classified lanthipeptides by sequence similarity network in various subtypes, thus identifying > 8000 members of all the four known classes of lanthipeptides. The extensive lanthipeptide analysis done by the group proved that the divergence in the lanthipeptide classes is ancient and supports the hypothesis that the lanthipeptide synthetases of different classes may have evolved through convergent evolution.

We utilized the phylogenetic distribution of RosM homologs as a guide to explore the evolution of roseocin. All the roseocin homologs identified using this approach lacked the already known lipid II binding motif (Grein et al. 2019) but were found to have a novel conserved motif in the two peptides. Considering that a naturally selected, more potent roseocin homolog may exist in nature, we selected the mutations that should not alter the Rosα peptide thioether rings, but could significantly influence its activity. Only the site-specific mutants were generated by an efficient one step site-directed single site plasmid mutagenesis protocol, followed by their successful PTMs by RosM in vivo. The semi-in vitro reconstitution (SIVR) strategy established earlier (Singh et al. 2020) was followed to obtain the bioactive core peptides for the determination of minimum inhibitory concentration (MIC) and structural characterization by mass spectrometry. Our study incorporated multiple approaches for investigating the lanthipeptide evolution i.e., an analysis of evolution rates between BGC types, analysis of variability within core peptide sequences versus leader peptide sequences, and an assessment of whether horizontal gene transfer is a major source of diversity. Assuming the BGC as a single evolutionary unit, many overlooked aspects of lanthipeptide evolution are revealed here that can aid the evolution-based studies of lanthipeptides in future.

## Materials and methods

### Genome mining for RosM like LanMs and phylogenetic analysis

RosM sequence (WP_010071701.1) was used as a query for blastp search on NCBI Genbank non-redundant protein sequences database (May, 2020). The top 100 hits were aligned with MUSCLE (Edgar 2004) using MEGA X (Kumar et al. 2018) and processed by GBlocks ver0.91.1 (Talavera and Castresana 2007) to select conserved domains for phylogenetic analysis by Bayesian method (Huelsenbeck and Ronquist 2001), available on an open server NGPhylogeny.fr (Lemoine et al. 2019). The phylogenetic tree was scrutinized to eliminate redundant hits from the same clade and genus. The remaining hits were pruned and analyzed using the maximum likelihood (ML) method and JTT matrix-based model with 500 bootstrap values in MEGA X using an outlier group. Homologs of RosM in Archaea (taxid:2157) were placed as the outlier group. The final phylogenetic tree was presented with iTOL v4 (Letunic and Bork 2019).

### Precursor peptides identification and biosynthetic gene cluster (BGC) analysis

The genome sequence of the hits was obtained in FASTA format from the Genbank database and analyzed with BAGEL4 (Hart and Moffat 2016) and antiSMASH 5.0 (Blin et al. 2019) web servers. Precursor peptides were identified manually by subjecting intergenic regions to NCBI ORF finder. Putative functions were confirmed based on CDD (Lu et al. 2020) and TMHMM server analysis (https://services.healthtech.dtu.dk/service.php?TMHMM-2.0).

### Tanglegram of evolutionary trees

Available 16S rRNA sequence was obtained from SILVA reference database SSU r114 (Quast et al. 2013) to plot the species tree. Nucleotide sequences of *lanM* and *lanA* were fetched from the Genbank. The species and gene tree were constructed with Maxiumum Likelihood (ML) method and subjected to the tanglegram algorithm (Scornavacca et al. 2011) incorporated in Dendroscope ver. 3.7.2. Species vs gene tree tanglegram was used to find horizontal gene transfer (HGT), while gene vs gene tree was used to test the coevolution of LanM and leader or core peptides in the same operon.

### Variability analysis

Variability among the set of genes was visualized in colour gradient matrix or graphical form using the sequence demarcation tool version1.2 (SDT v1.2) (Muhire et al. 2014). Shannon entropy for sequences aligned with clustal omega was calculated using a protein variability server (Garcia-Boronat et al. 2008). Evolutionary pressure (purifying, neutral, or positive) was tested using the Nei-Gojobori method in a codon-based Z-test of selection in MEGA X. The only sample under purifying or neutral selection (*p* value < 0.05) was considered for the next step to calculate the substitution rate (ῳ = d_N_/d_S_, i.e., the ratio of the rate of nonsynonymous substitutions (dN) and rate of synonymous substitutions (dS) per site in YN00 of PAML package (Pamilo and Bianchi 1993; Yang 2007).

### Variants generation by site-directed mutagenesis

Site-directed mutagenesis of selected roseocin alpha precursor peptide residues was carried out using Agilent's QuikChange site-directed mutagenesis kit. Primers and methodology were designed using the procedure described by Liu and Naismith 2008 study (Additional file 1: Figure S11). Desalted primers at a 0.05 µM scale were obtained from Sigma Genosys. The T_*m pp*_ (melting temperature of the primer-primer overlapping region) and T_*m no*_ (melting temperature of non-overlapping primer region) were calculated for each primer (Additional file 1: Table S7). PCR was carried out using Bio-Rad MyCycler thermocycler. The PCR products were treated with 5 units of DpnI at 37 °C for one hour, and the reaction was stopped by heating at 75 °C for 15 min. The PCR product was transformed into chemically competent *E. coli* DH5α. The mutations were confirmed through Sanger sequencing, and the plasmid was retransformed into *E. coli* BL21(DE3) for protein over-expression and in vivo post-translational modification by RosM (Singh et al. 2020), as per the details mentioned in Additional file 1.

### Reduction and alkylation of peptides

For confirming ring formation and availability of free cysteine(s), endoproteinase GluC (NEB, #P8100S) cleaved peptides (30 µM) in 50 mM Tris–HCl pH 8.0 were incubated with 1 mM TCEP (Tris(2-carboxyethyl)phosphine) at 37 ºC for 30 min to reduce the disulfide bond. Following reduction the peptide was alkylated by the addition of 10 mM IAA (Iodoacetamide) for 90 min. Reaction was set up in 60 µL volume in 1.5 mL MCTs. The samples were desalted using Pierce™ C18 spin column (#89873) before sample analysis with MALDI-TOF MS.

### MALDI-TOF MS analysis

Matrix-assisted laser desorption/ionization time-of-flight mass spectrometry (MALDI-TOF MS) was carried out on AB Sciex TOF/TOF 5800 system maintained at CIF, NABI, Mohali. The proteolytic digest was processed with Pierce™ C-18 spin column and mixed with α-cyano-4-hydroxycinnamic acid (1 mg/mL) for analysis in the reflectron mode. The mass spectra were calibrated using a mass standards kit for the calibration of AB scion TOF/TOF™ instruments (#4333604). The TOF/TOF explorer was used for data acquisition, and SeeMS (Chambers et al. 2012) and mMass programs (Niedermeyer and Strohalm 2012) were used for data analysis. To get a deep insight into the thioether rings pattern, we did MS/MS analysis for all the variants.

### Antimicrobial activity analysis

The minimum inhibitory concentration of wild-type roseocin and its variants was determined with micro broth-dilution method. Sterile 96-well microtiter plates were treated with 200 µL of 1% (w/v) bovine serum albumin (BSA) in 1 × phosphate-buffered saline (PBS) solution at 37 ºC for 30 min. After this, wells were washed with 1 × PBS to remove excess BSA (Ellis et al. 2020). Log phase culture was diluted with Mueller Hinton Broth (MHB) to obtain 2 × 10^5^ CFU/mL. The bacterial suspension and the lantibiotic were added to 96-well microplates at a ratio of 1:1. The microplates were incubated overnight at 37 ºC. The lowest concentration at which no visible growth was observed, was considered MIC.

## Results

### Phylogenetic analysis for selection of diverse RosM homologs

RosM (WP_010071701.1) installs thioether rings on two peptides (Rosα and Rosβ) that differ structurally and functionally but display synergistic antimicrobial activity as roseocin. Due to the unique natural promiscuity of RosM, it was speculated that RosM may have evolved distinctively and hence was subjected to query search in the GenBank database, which resulted in hits from a wide range of *actinobacteria* species and four other phyla (Additional file 1: Figure S1A). In the top 100 hits, a sequence identity ranging from 33.8–99.0% and conservation in CHG motif in the active site was observed. The majority of the hits represented *actinobacteria* (n = 45) and *cyanobacteria* (n = 29) (Additional file 1: Figure S1A). As expected, amino acid sequence identity criteria for genome mining generated an uneven distribution of hits, making it challenging to evaluate all the hits for novel BGCs. However, a Bayesian phylogenetic analysis of the obtained hits led to the phylum-wise clade formation along with the formation of subclades having BGCs of similar properties (Additional file 1: Figure S1B), thus helping in the systematic evaluation of distantly related RosM homologs. Interestingly, the RosM query search did not result in any hits from *firmicutes*, the only phylum with the lacticin 3147-like two-component lantibiotics discovered so far (Zhang et al. 2012). This observation indicated the independent evolution of roseocin family from lacticin family two-component lantibiotics.

**Fig. 2:**
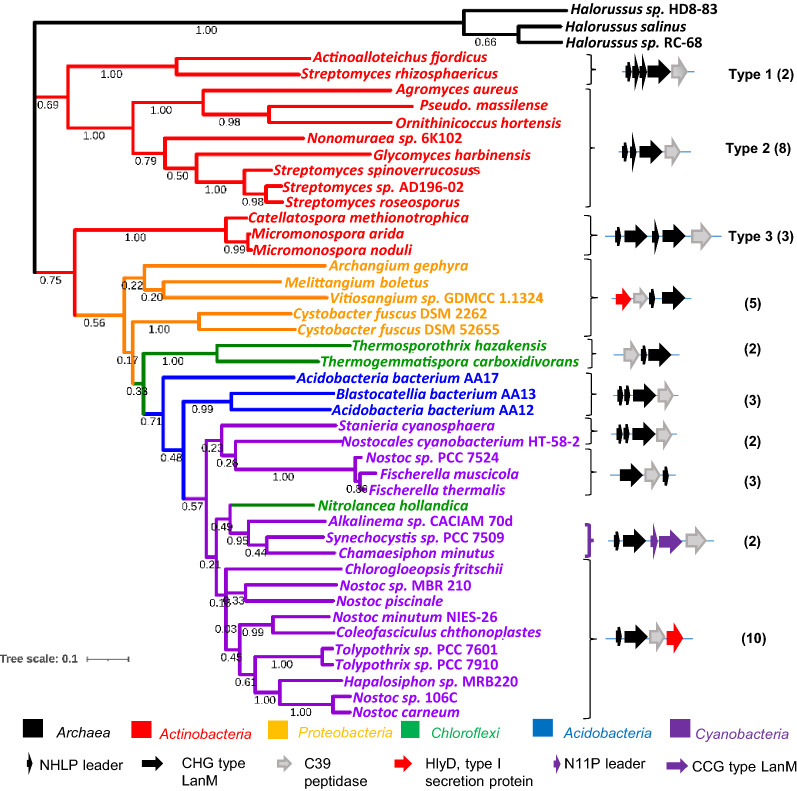
Phylogenetic tree of 42 selected RosM homologs showed conservation of gene locus and characteristic features along the phylogenetic tree. Roseocin family constituted the BGCs from *actinobacteria*, having three types of BGCs’ organization (type 1–3), each forming a separate subclade. CHG motif LanM for processing of NHLP type leader sequence was found in all the BGCs except in a subclade of *cyanobacteria* (*Synechocystis* sp. PCC 7509 and *C. minutus*) where conservation of two types of LanMs (CHG and CCG motif) and two types of leader sequence (NHLP and N11P) were found in a single BGC. LanMs from the BGCs of *Halorussus* genus were placed in the root. Value from 500 replicates bootstrap test is indicated on each branch. The numbers given in the bracket are the number of members of that particular subclade. *NHLP* nitrile hydratase leader peptide, *N11P* Nif11 derived peptides

To understand the features of the respective BGCs, we analyzed the genome sequences of RosM hits with BAGEL 4 (Hart and Moffat 2016) and antiSMASH 5.0 (Blin et al. 2019) webservers. Both software identified the BGC cluster boundaries including all the major genes of the BGC, but showed limitations in defining the genes encoding precursor peptides. Hence, we located the putative precursor peptide genes on the GenBank file or subjected intergenic gap regions to NCBI ORF-finder, enabling us to identify the specific precursor peptide encoding genes. As a major advantage of mining in a phylogeny-guided manner, identical lanthipeptide precursors (termed redundant hits) were easily identified in an initial analysis across the BGCs of the same subclade. For example, in BGC analysis across *actinobacteria*, 18 out of 22 hits from the *Streptomyces* genus and 10 out of 12 species from the *Micromonospora* genus encoded an identical precursor (Additional file 1: Figure S1B). Such redundant hits were eliminated to limit the sample size and prevent skewing the final sequence alignment. Phylogenetic branch lengths were observed as < 0.05 in LanMs of *actinobacteria* which corresponded to BGCs encoding identical precursors (Additional file 1: Figure S1). Hence, such RosM hits were removed from the rest of the phyla. Finally, 42 RosM homologs from five phyla (Additional file 1: Table S1) were selected for phylogenetic analysis using an appropriate outgroup for rooting. Unrooted trees, like in Additional file 1: Figure S1B, are only useful for visualization of the relatedness of sequences of different clades, while only a rooted tree provides insight into evolution. A careful selection of outgroups was followed, as suggested by Adamek et al. 2019, being neither too distant nor too close to the ingroups of the dataset under study. In a recent genome mining study (Makarova et al. 2019), archaea have been shown to contain lanthipeptide BGCs across the species of the *Halorussus* genus. Interestingly, these archaeal lanthipeptide BGCs are of class II type with a single CCG motif LanM and an unknown class of lanthipeptides. We selected three BGCs from the *Halorussus* genus in the archaea database and placed them as the outgroup to plot the maximum likelihood (ML) phylogenetic tree using a 500 bootstrap value (Fig. 2).

BGC analysis of each of the 42 RosM hits from the final phylogenetic tree (Additional file 1: Figure S2) showed a gradual shift in the genomic location of minimally required biosynthetic genes, *lanA* (lanthipeptide precursor), *lanM* and a bifunctional *lanTp* (peptidase domain-containing transporter) alongside the subclades (Fig. 2). In most BGCs, precursor genes were found upstream to *lanM*, which probably is the natural temporal order of their synthesis. The common observed feature in most of the precursor peptide genes was the NHLP (nitrile hydratase leader peptide) family signature (Haft et al. 2010) in their leader region and a single *lanM* gene of CHG-type present for their processing (Fig. 2). But more than one *lanM* genes carrying BGCs were also found across *actinobacterial* and *cyanobacterial* species. In *cyanobacteria*, these BGCs showed precursor peptides having conservation in leader regions from two divergent types of leader families, i.e. NHLP and N11P (Nif11 derived peptides) family, discussed later in detail (Fig. 4). Overall, these conservations and variations made it intriguing to study the dataset further for the conserved features of lanthipeptide evolution.

### Roseocin family BGCs identified in *actinobacteria*

**Fig. 3:**
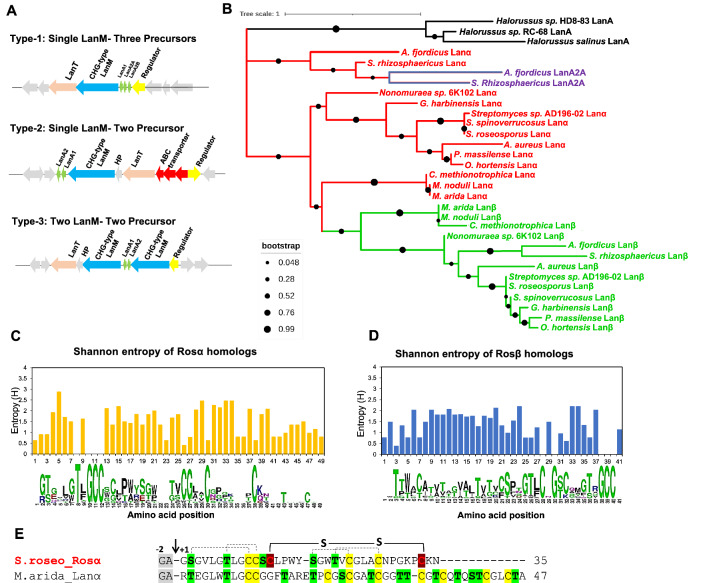
Diversity among the 13 representative members of the roseocin family. **A** Three common types of BGCs encode roseocin homologs, type-1, type-2, and type-3 BGC examples are of *S. rhizosphericus, S. roseosporus* NRRL 11379 and *C. methinotrophica*, respectively. **B** Phylogenetic tree of lanthipeptide core sequences with ML method with bootstrap values of 500 replicates. The exceptional third precursor (LanA2A) of the single LanM-three precursor i.e. type-1 BGC, is phylogenetically related to alpha peptides. Colour coding in Fig. 3B is red: alpha peptides; green: beta peptides; blue: third precursor core region. **C** and **D** Variation in the core peptide sequences as a function of Shannon entropy in the roseocin alpha and beta homologs, respectively. The alpha peptides contain a S/TxxxxTxGCC motif at the N-terminal end, and beta homologs contain a GS/TxxxxS/TxGCC motif at the C-terminal end. **E** A gigantic Rosα homolog from the *Micromonosporaceae* family contains nine Cys and thirteen Ser/Thr residues that may form as many lanthionine rings. Rosα of *Streptomyces roseosporus* (*S. roseo*) contains an indispensable disulphide bond and four (methyl)lanthionine rings (dotted lines depict the proposed ring topology in Rosα, Singh et al. 2020); *M. arida-Micromonospora arida*. *LanA* precursor peptide, *LanM* modification enzyme, *HP* Hypothetical Protein, *LanT* dual function peptidase-domain containing transporter

*Actinobacteria* showed the presence of three divergent subclades (Fig. 2), each displaying a characteristic pattern of genetic arrangement within the BGC. Initially, BGCs seemed unrelated owing to a difference in the organization of genes, sequence, and the number of lanthipeptide precursors, with some encoding more than one CHG-type LanMs (Fig. 2). However, further analysis showed that all BGCs encode precursor peptides homologous to either Rosα or Rosβ (Additional file 1: Figure S3A and B, respectively). Based on the chronological order in the phylogenetic tree, these 13 BGCs were classified as type 1–3, to represent their respective subclades (Figs. 2 and 3A). Roseocin was grouped as a member of the type 2 class, having stringent conservation of roseocin BGC features among the other mined members of the same subclade. Precursor peptides of type 2 BGCs also display features that agree with the earlier postulated structure of roseocin (Singh et al. 2020) and hence were used as a platform for designing the variants of Rosα (explained in later sections). However, precursor peptides in type 1 and 3 BGCs showed a more significant variation in the amino acids that might result in a different ring topology of these lanthipeptides (Additional file 1: Figure S3). The type 1 BGCs containing three instead of two precursor genes deviate from the usual two-component lantibiotics (Fig. 3A). Such kind of BGCs have been characterized earlier in the lacticin 3147 family (Xin et al. 2016; Zhao and Van Der Donk 2016). There, additional precursor was found to be a result of the duplication of one of the two genes. Contrary to this, we did not observe any core sequence similarity in the third precursor peptide of type 1 subclade (designated as LanA2A) to either of the other two lanthipeptides (Additional file 2), which ruled out an evolutionary gene duplication event. Phylogenetic analysis showed that LanA2A is closely related to alpha homologs (Fig. 3B). This indicates that LanA2A is either an alpha peptide that synergizes with a common beta-peptide, or it may be a constituent of a novel three-component synergistic system. As an advantage of random genome mining for lanthipeptide, many small-sized roseocin homologs were also found in the study by Walker et al. 2020 (Additional file 2). To classify such small-sized homologs into type 1–3 subclade, BGC analysis was done in the current study (Additional file 1: Figure S5) and various peculiar attributes like missing or duplicated genes, multiple LanMs, etc. were noted and hence, they could not be categorized as either of the members of type 1–3 subclade. These genes probably might be of lower significance and might have come into temporary existence to get eliminated during the natural selection for the most potential genes.

The type 3 BGCs of roseocin family, consisting of two LanMs and two precursor peptides (Fig. 3A), were confined to the *Micromonosporaceae* family (Fig. 2). These BGCs encoded a supersized homolog of Rosα, with the highest number of thioether-forming moieties (13 Ser/Thr and 9 Cys residues) in a single precursor peptide (Fig. 3E; Rosα has 5 Ser/Thr and 6 Cys residues). Such a huge precursor peptide probably necessitates a dedicated LanM for efficient post-translational modification in parallel to the LanM for the beta peptide. High pairwise sequence identity of LanMs of the *Micromonosporaceae* family (~ 50%) (Additional file 1: Table S2) indicates that this separate LanM might have resulted from a recent LanM gene duplication event (Additional file 1: Figure S4), unlike the two LanMs in the lacticin 3147 family, which have low sequence identity (24–29%) and one LanM has evolved specificity for modification of only one of the two precursors (Mcclerren et al. 2006). A similar sequence identity score in the pairwise alignment of lanthipeptide leaders (Additional file 1: Table S2) is surprising and can make sense only under the coevolutionary phenomenon, a perspective discussed in detail in the following sections.

As discussed earlier, alpha peptide initiates the interaction with the bacterial membrane by targeting lipid II, a key step in the mechanism of action of two-component lantibiotics (Bakhtiary et al. 2017; Oman et al. 2011). Most of the alpha peptides characterized to date possess an Asp/Glu residue containing lipid II binding motif (CTxTxD/EC), which is absent in Rosα peptide (Singh et al. 2020). Using the knowledge generated in the current study on the diversity of the roseocin family, it seems necessary to look for a novel motif for a similar or divergent action mechanism. To understand the variability and conservation of amino acid substitutions among all the roseocin homologs, a Shannon entropy (SE) analysis was done. Lower SE value (< 2.0) indicates higher conservation of amino acid residues through evolution (Garcia-Boronat et al. 2008). A conservation of a ten amino acid long stretch, S/TxxxxTxGCC, at the N-terminus of Rosα homologs (Fig. 3C) and an 11 amino acid stretch, GS/TxxxxS/TxGCC at the C-terminus of Rosβ homologs (Fig. 3D) was observed. Both the motifs were proposed to have a structure with overlapping lanthionine rings in our earlier study (Singh et al. 2020). Such a ring structure at the N-terminus of Rosα homologs is analogous to the nisin-like peptides (having two N-terminal rings, proven to be responsible for target binding), instead of an Asp/Glu residue-specific target binding motif of the two-component lacticin 3147-family lantibiotics (Cooper et al. 2008; Bakhtiary et al. 2017). Increased SE (> 2.0) in the other amino acid sequence positions (Fig. 3C, 3D, and Additional file 1: Figure S3A, S3B) revealed the innumerable combinations experimented by nature, as is evident by the changes in the number of Ser/Thr and Cys residues of the core region among the Rosα and Rosβ homologs. Except for the stretches mentioned above, substitutions were allowed at all the amino acid positions. Further, plausible exchange of indispensable disulfide of Rosα with thioether ring in *Streptomyces rhizosphaericus* (Additional file 1: Figure S3A); exchangeable lanthionine (Lan) and methyllanthionine (MeLan) rings; insertion/deletion of one or more thioether rings suggests the enormous scope of modular engineering of both, Rosα and Rosβ peptides (Additional file 1: Figure S3). The presence of a conserved motif and variability in the rest of the core region probably results from balanced combinatorial chemistry, operating parallelly with the conserved motif-oriented evolution of lanthipeptides.

However, the rest of the BGCs from other phyla showed no significant core sequence conservation. The lanthipeptides of *proteobacteria*, *chloroflexi*, *acidobacteria*, and *cyanobacteria* phyla seldom have significant antimicrobial activity (Mohr et al. 2015; Cubillos-Ruiz et al. 2017; Bothwell et al. 2021). Nevertheless, we proceeded further and discovered many overlooked aspects of lanthipeptide BGCs, providing new insights into lanthipeptide evolution.

### A new diversity-oriented class of lanthipeptides in *cyanobacteria*

**Fig. 4:**
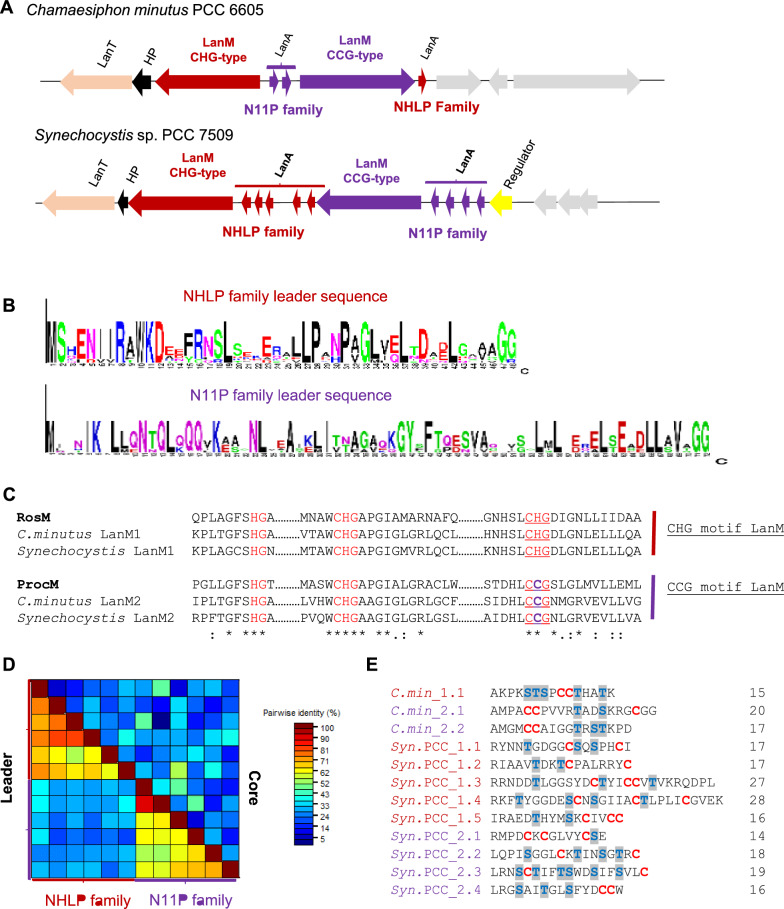
Novel BGCs, encoding diverse lanthipeptide core sequences, consist of two LanMs for processing two types of precursor peptides. **A** Two BGCs encoding NHLP and N11P family lanthipeptide leaders in their precursor peptides with the corresponding synthetases, i.e. CHG motif and CCG motif LanM, were identified in *Synechococcales*. **B** Sequence logos of NHLP family and N11P family lanthipeptide leader sequences using the precursor sequences from the above two BGCs. **C** Sequence alignment of cyclase domain of putative LanMs from characteristic BGC of *Synechococcales* showed a difference in catalytic motif. RosM like LanMs has a CHG motif, while ProcM like LanMs have a CCG motif. **D** Sequence identity percentage in the pairwise alignment of the 12 lanthipeptide precursors’ leader sequences (lower half) and core sequences (upper half). Diversity among lanthipeptide core sequences was high, irrespective of leader conservation. **E** Multiple sequence alignment of lanthipeptide core sequences depicts natural diversity. *LanA* precursor peptide, *LanM* modification enzyme, *HP* Hypothetical Protein, *LanT* dual function peptidase-domain containing transporter

Unlike significant conservation observed above in the core region of the roseocin-like lanthipeptides, diversity-oriented evolution is characterized by the generation of a vast variety of lanthipeptide core sequences with no conservation at all (Zhang et al. 2012; Cubillos-Ruiz et al. 2017). So far, prochlorosin-like BGCs are the only example which have evolved a highly promiscuous LanM (with CCG motif) for the maturation of diverse lanthipeptide sequences in marine *cyanobacteria* i.e. *Synechococcus* and *Prochlorococcus* (Li et al. 2010; Mukherjee and Van Der Donk 2014). Similarly, in our dataset, freshwater cyanobacterium species from *Synechococcales* also showed the diversity-oriented lanthipeptide BGCs, but with a novel, exquisitely divergent mechanism (Fig. 4).

In the current study, despite the expansion of hits across different phyla, obtained BGCs had CHG-type LanM, for processing NHLP-type lanthipeptide precursors (Fig. 2). NHLP family (or nitrile hydratase leader peptide; cl22942 subfamily TIGR03898) and N11P (Nif11 derived peptides; cl06756 subfamily TIGR03798) are the two well-characterized lanthipeptide leader types that have evolved from nitrile hydratase enzyme and Nif11 proteins, respectively (Haft et al. 2010). Usually, a single type of lanthipeptide leader, i.e. either of the NHLP or N11P, is observed in a BGC (Zhang et al. 2014). However, an exception was observed in the *cyanobacteria* (Fig. 2), which earlier were the source of prochlorosin family lanthipeptides as well (Cubillos-Ruiz et al. 2017). A non-conventional BGC with both types of lanthipeptide leader (NHLP and N11P), along with two LanMs in a single BGC, for the maturation of three and nine precursor peptides (Fig. 4A and B) was identified by a manual search of the nearby ORFs. This type of BGC was found confined to *Synechococcales* and included *Synechocystis* sp. PCC 7509 and *Chamaesiphon minutus* as member species (Fig. 4A). As N11P family lanthipeptides are only associated with the CCG motif LanM, we speculated one of the LanMs to be the CCG motif LanM. Surprisingly, sequence alignment showed the presence of ProcM-like CCG motif LanM in the same BGC besides a CHG motif LanM (Fig. 4C). This unprecedented example of association between two leader types and two LanM types in a single BGC indicates another evolved mechanism of diversity-oriented BGCs in *cyanobacterial* species that could be a better way of efficient biosynthesis of diverse lanthipeptide core sequences (Fig. 4D and E).

Three lanthipeptide precursor sequences (2 + 1) in *C. minutus* and nine (4 + 5) in *Synechocystis* sp. PCC 7509 represents an intermediate number of diverse sequences observed earlier for prochlorosin-like genes (Cubillos-Ruiz et al. 2017). A truncated gene found in *C. minutus* genome (Additional file 2) could result from mutations like frameshift or early stop codon, preventing the synthesis of a functional ORF. Such pseudogenes are a common feature of the prochlorosin family lanthipeptides and support the ongoing diversification of precursor genes in a diversity-oriented manner (Cubillos-Ruiz et al. 2017). In the *C. minutus* genome*,* four more distantly located N11P-type lanthipeptide precursors were found, which might also be associated with this BGC (Additional file 1: Figure S2). Intrigued by the novel mechanism of diversity generation in *Synechococcales*, we further analyzed the other BGCs to identify conserved features of relevance.

### Coevolution of lanthipeptide leader and lanthipeptide synthetase among different phyla

**Fig. 5:**
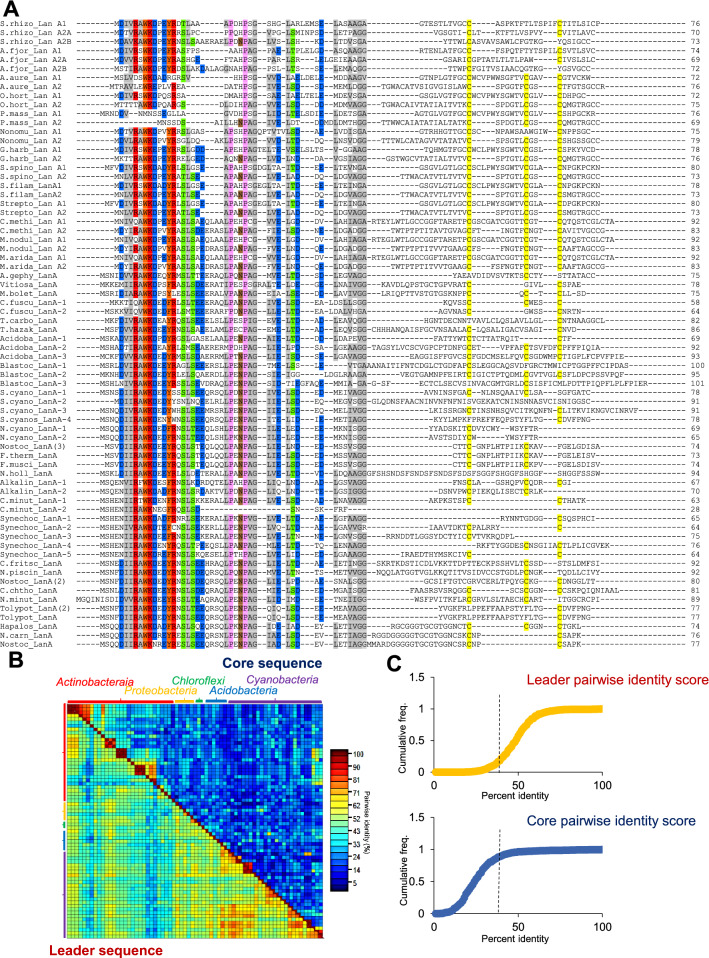
Conservation of lanthipeptide leader sequence over diverse core sequences. **A** Multiple sequence alignment using MUSCLE of all the identified 68 precursor peptides from 42 BGCs. All leader sequences are from the NHLP family of lanthipeptide leaders. Conserved residue positions are highlighted. **B** Pairwise identity among 68 lanthipeptide leader sequences (lower half) and core sequences (upper half) showed high similarity among leader over core sequences (except lanthipeptide core sequences of the roseocin family having core conservation). **C** Cumulative frequency of pairwise sequence identity among the lanthipeptide leader and core sequences, respectively. 80% of lanthipeptide leader pairwise alignment showed > 39% identity. However, only 10% of lanthipeptide core pairwise alignment fulfilled the same criteria (mainly of the roseocin family)

Lanthipeptide precursor is derived from an assimilation of a protein tailored as a leader sequence with an independently evolving core sequence rich in Ser/Thr and Cys residues (Haft et al. 2010; Zhang et al. 2012). In a previous study by Zhang et al. 2014, the ProcM (having CCG motif) was used for genome mining, and thus obtained BGCs showed highly varying lanthipeptide leader families. However, in our study, despite the diversity among BGCs from a wider range of phyla, high conservation among the leader region of the precursor peptides was observed (Figs. 2 and 5A). The only exception was *cyanobacterial* species (Fig. 4), which could be unearthed by manual inspection of ORFs that otherwise would have been missed (Singh and Sareen 2014; Zhang et al. 2014). Thus, we found that all leader peptide sequences belonged to the NHLP family (Fig. 5A). In 80% of pairwise sequence alignments of leader sequences, we observed > 39% sequence identity; while only in 10% of the core sequences pairwise alignments, an identity of > 39% was observed (Fig. 5B and C). These identity scores support the fact that conservation in leader peptides does not restrict the lanthipeptide core diversification even among different phyla. Variability pattern was also plotted for all the 42 LanMs (Additional file 1: Figure S6A) which surprisingly had an overlap with the variability in leader regions (Additional file 1: Figure S6B). Earlier, the conservation of two leader family types and two different types of LanMs in a single BGC of *C. minutus* and *Synechocystis sp.* (Fig. 4) also suggested an essential linkage between the leader and lanthipeptide synthetases.

**Fig. 6:**
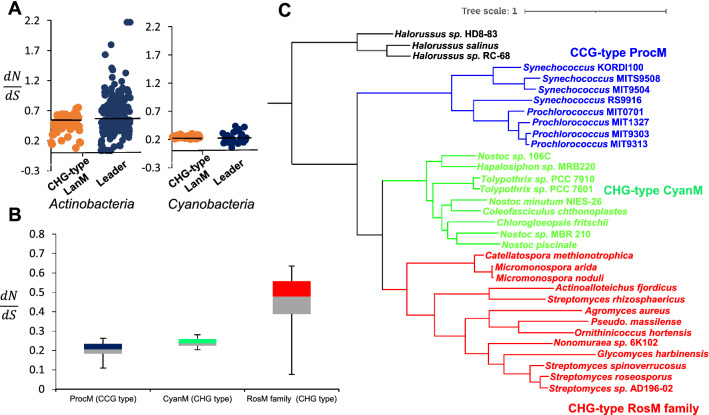
Coevolution of lanthipeptide leader and synthetase in a phylum-dependent manner. **A** Pairwise d_N_/d_S_ distribution of lanthipeptide leader and *lanM* from *actinobacteria* (13 BGCs of roseocin family) and *cyanobacteria* phylum (9 BGCs) showed variation in different phyla suggesting coevolution of the lanthipeptide leader and synthetases. **B** Standard box plot of the evolutionary rates of CCG motif prochlorosin family LanMs (Cubillos-Ruiz et al. 2017) (Additional file 1: Table S3) and CHG motif LanMs of *cyanobacteria* (from the current study) showed a similar pattern (median 0.21 and 0.24, respectively), while the CHG motif *lanMs* of roseocin family in *actinobacteria* has a higher value (median 0.48). In the standard box plot, the lower and upper shows the first and third quartile values, respectively, separated by the median value. The error bar plots the minimum and maximum values. **C** Phylogenetic tree of CCG motif ProcMs (Cubillos-Ruiz et al. 2017) and CHG motif LanMs of *cyanobacteria* and *actinobacteria* (from current study). Prochlorosin family LanMs displayed significant divergence from CHG motif LanMs of *cyanobacteria* and *actinobacteria*

**Fig. 7:**
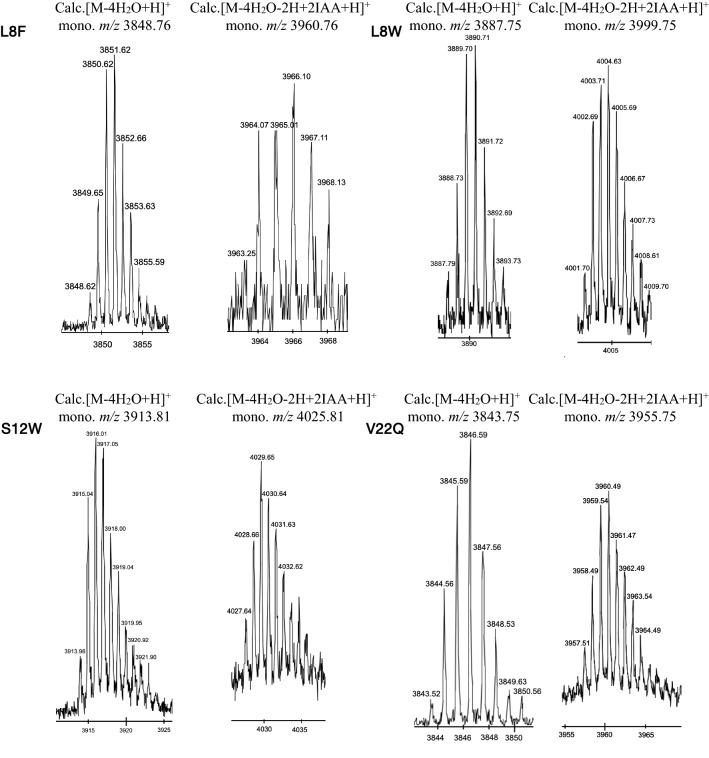
MALDI-TOF MS of endoproteinase GluC treated Rosα after TCEP only and after TCEP + IAA alkylation assay. Additional file 1: Table S5 represents the calculated and observed mass of the respective species of each variant generated

To explore further, we determined the mutation rates of both the *lanM* and the lanthipeptide leader genetic region by calculating the d_N_/d_S_ ratio, which is the ratio of the rate of nonsynonymous to synonymous mutations. The *lanM* and lanthipeptide leader genetic regions from 13 BGCs of *actinobacteria* (roseocin family) and 9 BGCs of *cyanobacteria* were selected for the separate analysis of two phyla. The calculated d_N_/d_S_ for *lanM* and leader peptide exhibited distinct patterns for the two phyla (Fig. 6A). The d_N_/d_S_ ratio was in agreement with the phylum-wise evolution of lanthipeptide synthetases (Zhang et al. 2012), as well as the coevolution of lanthipeptide synthetases and leader sequences. It has been proposed earlier by Cubillos-Ruiz et al. 2017, that a lower d_N_/d_S_ ratio is confined only to the *lanM*s having the CCG motif of the prochlorosin family (or ProcMs), suggesting an evolutionary locked state that favors the catalytic promiscuity for the processing of diverse precursors. Interestingly, *cyanobacterial lanM*s with CHG motif (hence we proposed the name, CyanMs) found in the current study also displayed a lower d_N_/d_S_ ratio, i.e. 0.24 (Fig. 7B), suggesting an evolutionary linkage between CyanMs and ProcMs. However, phylogenetic analysis showed a significant divergence of ProcMs from CyanMs (Fig. 7C) even with similar d_N_/d_S_ values (Fig. 7B). This indicates that lanthipeptide synthetases of the *cyanobacteria* must have diverged during evolution into two subclades of CHG and CCG motif LanMs, both being locked into a similar evolutionary conserved state and probably having a similar level of substrate tolerance. The reason for such a divergence is not clear, but the significance of phylum in deciding the fate of lanthipeptide synthetases enforces the phylum-dependent effect on the evolution of lanthipeptides than proposed earlier (Cubillos-Ruiz et al. 2017).

### Production of evolutionarily selected natural variants of Rosα

The gene-encoded nature of lantibiotics offers an opportunity to bioengineer the peptide components for obtaining roseocin variants with improved bioactivity/physicochemical parameters. The evolutionarily selected natural variants of Roseocin peptides helped us identify the structural regions amenable to amino acid substitutions. Keeping in mind the earlier common observation on substitutions that disrupt (methyl)lanthionine formation, ring size or lanthionine location leading to structural alteration with loss of bioactivity (Barbosa et al. 2019; Bédard et al. 2019; Field et al. 2015; Rahman et al. 2021), and the limitation of PTM enzymes that generally do not accommodate all amino acid substitutions (Cooper et al. 2008), only a few naturally allowed substitutions were tried to prove our concept.

We focused specifically on the *actinobacteria* clade of the roseocin family phylogenetic tree, as the sequences arising from BGCs sharing close common ancestors and, thus, the same mode of action are likely to group into the same clade. For generating naturally inspired roseocin variants, we identified the most conserved and divergent sites in roseocin peptides’ homologs (Fig. 3C and D). Variants were restricted to the type 2 subclade only (roseocin being its member; Fig. 3A), as types 1 and 3 exhibited significant divergence in BGC structure along with a divergence in the sequence of both alpha and beta components. Further, highly divergent variations of type 1 and 3 subclades from the roseocin peptides might hinder the post-translational modifications by RosM. A consensus sequence was obtained using the alignment of Rosα and Rosβ homologs from the type 2 subclade for the naturally permissible mutations (Additional file 1: Figure S7). As alpha peptide plays a pivotal role in the mechanism of two-component lantibiotics (Oman et al. 2011; Bakhtiary et al. 2017), Rosα peptide variants were designed first. Among Rosα homologs, essential amino acid residues required for (methyl)lanthionine rings installation showed high conservation along with the two cysteines involved in the disulphide bond formation. Another conserved stretch i.e. S/TxxxxTxGCC was clearly noticeable, which we speculate to be a putative target binding motif. Two critical observations favour this speculation: first, the ‘ring within a ring’ structure of this N-terminal region, proposed by us due to the absence of fragment ions in the MALDI-TOF MS/MS analysis (Singh et al. 2020). Second, from the pockets and mouth information revealed by the homology modelling of 37 characterized lantibiotics (Chakraborty et al. 2019), the disordered residues (the ones involved in protein–protein interactions or target binding) were found within the pockets/rings and hence are thought to undergo a transition from disorder to order upon target binding. We hypothesize that the four variable residues in this stretch (3-GVLG-6) probably indicate disorder among the fully conserved ring-forming residues. Hence, alterations in this motif can significantly affect the binding affinity of Rosα to its target and consequently the antimicrobial activity/MIC of roseocin. Leu8 of this motif seems to be significant due to its relatively high conservation, with the only naturally allowed substitutions being L8I, L8F, and L8W. Leu8 was thus mutated to F and W. Serine at position 12 being the residue that we characterized earlier to have escaped dehydration (Singh et al. 2020) and W being the only naturally allowed substitution, we tried substituting S12 for W. V22Q substitution was selected for it might affect the overall hydrophobicity of the peptide and hence its interaction with the target.

**Fig. 8:**
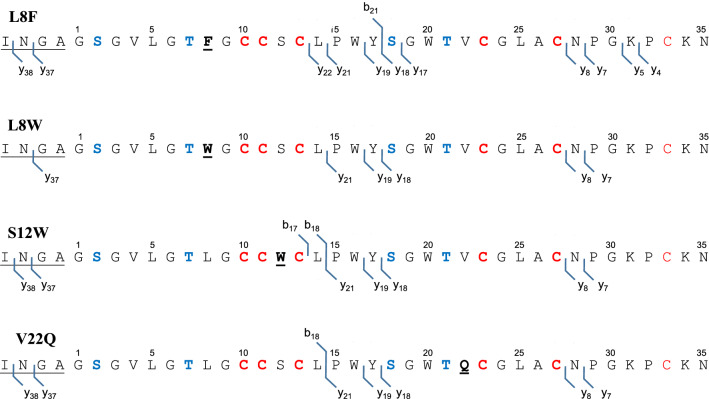
Tandem MS fragmentation pattern of alkylated Rosα variants confirms the similar ring topology in all the variants as that of wild-type Rosα, published earlier (Singh et al. 2020). Underlined regions correspond to leader sequence overhang that remained attached after endoproteinase GluC proteolytic digestion

These evolutionarily selected Rosα variants were generated by site-directed mutagenesis and tested for the PTM by RosM in vivo in *E. coli* BL21(DE3). Notably, unlike the other studies where low expression of the lantibiotic variants is often observed (Field et al. 2013; Geng and Smith 2018), phylogeny-guided mutations generated in Rosα led to a substantial yield of the bioengineered variants to allow their further analysis. All four RP-HPLC purified full-length peptide variants were analyzed by ESI–MS (Additional file 1: Figure S8; Table S4). As was reported earlier for the wild-type Rosα, that underwent four-fold dehydration (Singh et al. 2020), observed mass peaks in Rosα L8F, L8W, S12W, and V22Q agreed with the calculated mass having four-fold dehydrations and one disulphide bond (Additional file 1: Table S4). An additional peak of + 89 Da (double-charged ion for 178 Da) mass shift indicated the presence of species with N-terminal gluconoylation. The RP-HPLC purified peptide variants were subjected to leader cleavage with endoproteinase GluC and analyzed with MALDI-TOF MS and MS^n^ (Fig. 7). The MALDI-TOF MS data of the four variants (after TCEP treatment) agreed with the calculated m/z of the reduced peptide i.e. 2 Da higher than the theoretical monoisotopic mass, indicating a reduction of the disulfide bond between Cys13 and Cys33 (Fig. 7). Alkylation with IAA confirmed the disulfide bond and assessed the presence of free cysteines, if any, arising due to incomplete dehydration or cyclizations. A mass shift of 57 Da was expected per available free cysteine residue. It was observed that the natural variants were fully dehydrated and cyclized to possess four (methyl)lanthionine rings and a disulfide bond (Fig. 7, Additional file 1: Table S5). The MS^n^ analysis of the leader-cleaved alkylated peptides further revealed protection imparted by (methyl)lanthionine rings. The absence of b and y fragment ions for the stretches 2-SGVLGTLGCC-11 and 18-SGWTVCGLAC-27 confirmed thioether ring protected regions in all the variants (Fig. 8), similar to wild-type Rosα (Singh et al. 2020). The fragment ions (Additional file 1: Table S6) confirmed the dehydration of Ser2, Thr7, Ser18, Thr21 and the undehydrated status of Ser12. The mass shift of 57 Da in y4-y22 indicated the alkylation of Cys33 and a shift of + 114 Da in the fragment ion next to y22 the disulfide bond partner as Cys13. The observed fragment ions confirmed the presence of a disulphide and 4 (methyl)lanthionine rings in all the Rosα variants (Fig. 7). All the variants were tested for their bioactivity in combination with wild-type Rosβ by agar diffusion assay against *M. luteus* ATCC 10240, after leader cleavage by proteinase K (Additional file 1: Figure S9). Bioactivity was observed with all the variants (Additional file 1: Figure S9) however, minimum inhibitory concentration (MIC) was determined to compare the efficacy of variants with respect to wild-type Rosα against *M. luteus* ATCC 10240 and methicillin-sensitive *Staphylococcus aureus* (MSSA) ATCC 25923 (Table 1). Variants generated by substitution within the N-terminal conserved motif of Rosα i.e. L8F and L8W showed improvement in the MIC. RosαL8F exhibited four-fold lower MIC against MSSA ATCC 25923, while RosαL8W showed two-fold lower MIC against *M. luteus* ATCC 10240. However, no improvement with RosαS12W and a loss of potency in RosαV22Q were observed. Considering individual roseocin homologs have many simultaneous amino acid alterations instead of a single residue (Additional file 1: Figure S3), some mutations like V22Q might depend upon those other amino acids to exert a positive effect on antimicrobial activity. The MIC data shows that our strategy of mutants generation to obtain only the bioactive variants was successful and hence can be extrapolated to obtain multi-residue variants to match the natural congeners. The thus-engineered best variant can be subjected to detailed structural and functional characterization for development into a new drug.

**Table 1:** Minimal inhibitory concentration (MIC) of wild-type roseocin and its variants

Lantibiotic	MIC against *M. luteus* ATCC 10240 (µM)	MIC against MSSA ATCC 25923 (µM)
Roseocin (wild-type)	0.5	2
Roseocin L8F	0.5	0.5
Roseocin L8W	0.25	2
Roseocin S12W	0.5	> 16
Roseocin V22Q	> 8	> 16
Nisin	0.0625	2

### Phylogeny-based ring structure prediction.

Structure elucidation of lanthipeptides is often hindered owing to low yield and complexity in the NMR data acquisition and solving the data. Tandem MS has been widely successful in depicting the structure with a low amount of lanthipeptides. However, a lack of fragmentation is observed in the region with overlapping rings (Garg et al. 2012; Singh et al. 2020), like that in wild-type Rosα peptide and its variants (Fig. 8). The structural features of roseocin peptides could be solved only to the extent of alternative possibilities in the ring pattern of Rosα, while in Rosβ, only the first two rings A and B could be figured out by tandem MS. As an unanticipated advantage of the current phylogenetic study, the ring pattern in the overlapping regions of both the roseocin peptides could further be unfolded, as it seemed that they are dispensable, probably to gain some advantage in terms of antimicrobial activity after the ring alteration/structure modification.

Since (methyl)lanthionine rings are the result of bond formation between a conserved Thr/Ser and a Cys residue, the simultaneous deletion of both the Ser/Thr and Cys residues was used as the hallmark for deducing the ring pattern. Rosα peptide contains four (methyl)lanthionine rings (labelled A to D) and one disulfide bond. Based on earlier tandem MS study, Rosα is a globular-shaped peptide with two pairs of overlapping regions, i.e. N-terminal rings A and B, and the C-terminal rings C and D (Singh et al. 2020). Ring A and B are part of the essential motif of Rosα and its homologs; therefore, the mutation in this region was neither expected nor found in any of the homologs. However, in the C-terminal region, simultaneous deletion of constituent residue partners was observed in Rosα homolog from *Nonomuraea* (Additional file 1: Figure S10A)*,* showing the possible location of ring C.

Rosβ structure has been proposed earlier as a linear peptide with a total of six (methyl)lanthionine rings (labelled A to F). The rings (A and B) were identifiable by tandem MS (Singh et al. 2020), while the rest are part of the overlapping ring structure. Rings E and F seem indispensable, hence, their ring topology could not be predicted here. Ring C location has been predicted by missing constituent residue partners in *M. Muleris, A. radicidentis* etc. (Additional file 1: Figure S10B). This left only one possibility of ring D for which missing residue partners can also be confirmed in Rosβ homologs of *S. rhizosphaericus* and *A. fjordicus* (Additional file 1: Figure S10B).

## Discussion

Phylogeny-guided mining approach has been successfully applied to discover new NRPS/PKS natural products from microbial genomes and metagenomes (Kang 2017), but it is for the first time being applied here for two-component lantibiotics. Phylogeny-guided approach for mining roseocin family lantibiotics displays a comparative view to the genome mining study of Walker et al. 2020, which identified and classified > 8000 precursor peptide hits, including the members of the roseocin family. Cyclase domain (being a common feature among all the currently known classes of lanthipeptide synthetases) was used as a query [i.e. LANC_like (PF05147) hidden Markov model (HMM)] from the Protein family (Pfam) database (Finn et al. 2016), which resulted in 12,705 proteins. Further classification into Class I–IV lanthipeptide BGCs was achieved by analyzing the genomic content of each of these for the presence of Pfam HMM for the dehydratase domain/LanM/protein kinase (Class III and IV). Further on, genes for precursor peptides (being the most diverse and hence the least similar) were identified in a representative sample from the BGC of each class by manual examination of the lanthipeptide-specific features and genetic distance from the processing enzyme. Unlike our study, the phylogenetic distance and BGCs organization formed the basis of the grouping and subgrouping to understand lanthipeptide diversity. However, the proposed class II29/31 for Rosα homologs excluded roseocin family members of the type 3 subclade, from the *Micromonosporaceae* family while at the same time included many redundant hits of the roseocin family. For Rosβ homologs, class II2 was proposed that included hits from unrelated distant phyla, *firmicutes* and *cyanobacteria* (roseocin being a member of *actinobacteria*). Thus, the sequence logo generated from there, varies hugely in member species and conservation patterns to ours. However, a larger sample size in Walker et al. 2020 study helped in identifying five new BGCs of the type 2 subclade of the roseocin family (single LanM-two precursor BGC; Additional file 2), making type 2 the largest subclade of roseocin family (including eight from the current study) causing it to have total 13 members (Additional file 1: Figure S3). A consensus logo obtained by the multiple sequence alignment of these 13 members has formed the basis of our amino acid residue identification to generate Rosα variants (Additional file 1: Figure S7A). Overall, the combined approach of genome mining tools with the evolutionary principles can rapidly and conclusively classify the members of all the lantibiotic families, along with designing the natural variants to select candidates with better efficacy.

Recently, by coupling genome mining methods to identify evolutionarily related polymyxin family-like BGCs from ~ 10,858 sequenced bacterial genomes, Wang et al. 2022b successfully identified macolacin, a colistin-like antibiotic that is active against colistin-resistant Gram-negative pathogens. Since the colistin resistance is mediated by either *mcr-1* or intrinsic PEtN transferase genes (*eptA*), they reasoned that a solution might have evolved through natural selection to circumvent this troubling resistance mechanism and bioinformatically searched for the naturally evolved colistin congeners.

Using the phylogeny-guided genome mining approach, another nature-inspired lipopeptide antibiotic, cilagicin was discovered that exhibits a distinct mode of action (Wang et al. 2022a). Out of the ~ 10,000 sequenced bacterial genomes, a phylogenetic tree was constructed using sequences of condensation starter (Cs) domain that installs the N-terminal lipid in lipopeptides. The clades that fell out as a separate group were selected to identify cryptic BGCs, as a potential source of an uncharacterized lipopeptide antibiotic. The structure of the encoded product was bioinformatically predicted and chemically synthesized, thus producing a synthetic-bioinformatic natural product (syn-BNP), cilagicin. Phylogenetic analysis of lanthipeptide synthetases has formed the basis of our current study to identify roseocin family lantibiotics and therefore gene crossover events of lanthipeptide synthetases during evolution can be a significant determinant of the fate of lanthipeptides diversification in nature. Gene crossovers of lanthipeptide synthetases can be predicted using a tanglegram, which indicates a difference in gene topology (*lanM* in our case) *vis-à-vis* species evolution tree (16S rRNA). Thus, a comparative phylogenetic analysis of the 16S rRNA-based species tree with the *lanM* gene tree was plotted in the dendroscope (Scornavacca et al. 2011), allowing swapping of the branches from both of the trees at the possible closest distance and HGT events were inferred by crossover lines between the gene and species tree (Zhang et al. 2016). Remarkably, the final tanglegram showed only intra-phyla gene crossover events (Additional file 1: Figure S12), which supports the phylum-dependent evolution of lanthipeptide synthetases and hence a probable dispersion of the lanthipeptides within the same phylum. To check the BGC’s organization in correlation to the 16S rRNA from *actinobacteria* and *cyanobacteria* (while archaeal 16S rRNA was placed as an outgroup), two different ML phylogenetic trees were also plotted (Additional file 1: Figure S13). In the LanM-based phylogenetic tree, a systematic classification of the BGC’s structure was obtained for both *actinobacteria* and *cyanobacteria* (Fig. 2), which is not apparent in the 16S rRNA-based phylogenetic trees (Additional file 1: Figure S13). Again, the intra-phyla gene crossover events (Additional file 1: Figure S12) could be the precise reason for the difference in the BGC’s classification in the two kinds of phylogenetic trees.

The biosynthesis of a lanthipeptide involves the installation of thioether ring topology in a selective stereochemistry (Mukherjee and Van Der Donk 2014). The ring topology in the core sequence of a lanthipeptide seems to evolve as a result of the selection pressure of the target structure (Zhang et al. 2012). However, no similar evolutionary pressure over the leader sequence could be postulated. The role of lanthipeptide leader in interaction of the precursor peptide with lanthipeptide synthetase is known to impel the core and enzyme into a conformationally constraint structure for successful post-translational modifications (Li et al. 2017). Recent studies have confirmed the significance of the leader peptide in successful modification of cognate and noncognate precursor peptidesd by lanthipeptide synthetase LanM (Burkhart et al. 2017; Viel and Kuipers 2022). Findings, like a highly conserved lanthipeptide leader with diversity in the core peptide (Cubillos-Ruiz et al. 2017); vital conservation of FxLx motif in lanthipeptide leaders for class I lanthipeptide synthetases (Abts et al. 2013); chimeric leader peptide for post-translational modifications by two different classes of synthetases (Burkhart et al. 2017) and the unique layout of mersacidin leader for MrsM (Viel and Kuipers 2022) suggests the unique function of leader region in post-translational maturation of the bioactive core peptide. Our study adds an evolutionary logic by showing a coevolutionary relationship between lanthipeptide leader and lanthipeptide synthetase in a phylum-dependent manner (Fig. 6). Fewer crossovers in the tanglegram of phylogenetic trees of lanthipeptide leader and LanM sequences (Additional file 1: Figure S14), also point towards the coevolution of lanthipeptide leader and lanthipeptide synthetases to maintain the required interactions for post-translational modification in the core sequence. This observation opens up new possibilities for future combinatorial biosynthesis and emphasizes using the phylogenetically related pair of lanthipeptide leader and synthetase to produce novel cognate and non-cognate lantibiotics efficiently.

Some BGCs, identified here are the only representatives of their group that can be explored by in vitro studies for novel lanthipeptides. *Chloroflexi* and *acidobacteria* constitute bacterial species from extreme ecological conditions and the identified precursor peptides were found enriched with thioether-forming moieties (Ser/Thr and Cys) that, in addition to bioactivity may provide enhanced stability at extremes of temperature and pH (Additional file 1: Figure S2, Additional file 2), an important feature desired in industrial applications. Extremophiles can be a source for thermostable PTM machinery for bioengineering. Another important observed feature was the highly conserved dual transport secretion system of HylD membrane protein and the double glycine peptidase domain containing LanTp in *proteobacteria* and *cyanobacteria* subclades (Additional file 1: Figure S2). It has been proposed that HylD plays a vital role in exporting lanthipeptide across the outer membrane of Gram-negative bacteria (Haft et al. 2010). The dual transport system is a notable feature among BGCs identified in Gram-negative bacteria (Mohr et al. 2015).

Prochlorosin synthetase (ProcM)-like LanMs in *cyanobacteria* are a vital component of diversity-oriented lanthipeptide evolution due to its unparalleled capacity to process diverse types of lanthipeptide cores (Cubillos-Ruiz et al. 2017). However, in our dataset, freshwater cyanobacterium species from *Synechococcales* showed an association of ProcM-like LanMs (CCG motif) with RosM-like LanMs (CHG motif) within a single BGC suggesting a novel and exquisitely divergent mechanism of diversity-oriented evolution in lanthipeptides from *cyanobacteria* (Fig. 4). Further, a similar evolutionary rate among the LanM with CCG and CHG motif across the *cyanobacteria* phylum (Fig. 6B) increases the possibility of similar promiscuity in substrate tolerance. The two earlier genome mining studies on BGCs of *Synechocystis* sp. PCC 7509 and *Chamaesiphon minutus* missed the underlying association of two diverse leader types and LanM classes in a single BGC (Singh and Sareen 2014; Zhang et al. 2014), which strengthens the perspective of studies done in a phylogeny-guided manner to discover of novel lanthipeptides.

## Supplementary Information

**Additional file 1: Figure S1.** (A) Pie chart of the obtained hits, with most of the hits being from *actinobacteria* and *cyanobacteria* phylum. (B) Phylum-dependent clade formation of 100 RosM homologs in Bayesian analysis. **Figure S2.** Complete BGCs of selected 42 LanMs based on BAGEL 4 and antiSMASH 5.0 prediction. Annotation is based on CDD analysis. **Figure S3.** Multiple sequence alignment of roseocin family lanthipeptide precursor sequences to determine the conserved motif. **Figure S4.** Bayesian analysis of two LanMs of the same biosynthetic gene cluster from roseocin and lacticin 3147 families. **Figure S5.** (A) Distantly related BGCs of roseocin family from Walker et al. 2020 study, with multiple numbers of precursors in gene clusters. (B) Rosα homologs of these BGC are unusually short but contain the conserved motif S/TxxxxTxGCC. **Figure S6.** (A) Pairwise sequence alignment and percent identity of 42 LanM sequences (B) and the comparative cumulative frequency of identity percentage among leader, core, and LanM protein sequences of 42 BGCs. **Figure S7.** Sequence logo from multiple sequence alignment (A) Rosα and (B) Rosβ homologs from type 2 subclade showed highly conserved positions and possible sites of evolutionary variation. **Figure S8.** ESI-MS data showed that variants of Rosα were post-translationally modified by RosM, in *E. coli* BL21(DE3). **Figure S9.** Evaluation of antimicrobial activity in synergism with Rosβ after leader removal with proteinase K. **Figure S10.** Multiple sequence alignment of Rosα homologs to predict the ring topology in (A) Rosα and (B) Rosβ. **Figure S11.** Schematic representation of the primer design for site-directed mutagenesis. The arrowheads represent the site of mutation in the primer-primer overlapping region. **Figure S12.** A tanglegram between the 16S rRNA and the *lanM* gene tree indicates that intra-phyla horizontal gene transfer (HGT) is a major source of lanthipeptide diversity. **Figure S13.** Maximum likelihood phylogenetic tree of 16S rRNA from bacterial species of (A) *actinobacteria* and (B) *cyanobacteria*, in correlation of their BGCs organization. **Figure S14.** (A, B) Tanglegram between the phylogenetic tree of LanM and their associated lanthipeptide leader/core in Dendroscope. 4. **Table S1.** 42 selected hits from RosM search in the NCBI database. **Table S2.** A comparison of the roseocin and lacticin 3147 families of two LanM-two precursor genes. **Table S3.** Accession number of ProcM family LanMs from Cubillos-Ruiz et al. 2017. **Table S4.** Calculated (by Expasy) and observed (by ESI-MS) average mass of full length (with leader region) post-translationally modified Rosα-wild type and its variants. Selected positions for mutant generation are bold and italicized. **Table S5.** Calculated (by Expasy) and observed (by MALDI-TOF MS) monoisotopic mass of Rosα variants after leader cleavage using endoproteinase GluC (leaving an overhang of four amino acid residues) reduced with TCEP only & TCEP reduced peptides alkylated with IAA. **Table S6.** b and y ions obtained in MS-MS fragmentation of Rosα variants. **Table S7.** The list of primers used for the SDM-PCR in the generation of variants (F-forward and R-reverse primer).

**Additional file 2.** Amino acid sequence of lanthipeptide precursors associated with (A) 42 BGCs of Figure 2 (main text), and (B) additional roseocin homologs from Walker et al, 2020 study.

## Data Availability

Original raw dataset can be accessed in Mendeley dataset: https://data.mendeley.com/datasets/nbwzz4gg6v/draft?a=a6d56111-2ed8-4895-b578-47ff2c474bde..
