# Supplementary material for: Phylogeny-guided genome mining of roseocin family lantibiotics to generate improved variants of roseocin
Source: AMB Express. 2023 Mar 20;13:34. doi: 10.1186/s13568-023-01536-9 (PMC10027976; doi:10.1186/s13568-023-01536-9)
Supplement: Supplementary file 1 — Additional file 1: Figure S1. (A) Pie chart of the obtained hits, with most of the hits being from actinobacteria and cyanobacteria phylum. (B) Phylum-dependent clade formation of 100 RosM homologs in Bayesian analysis. Figure S2. Complete BGCs of selected 42 LanMs based on BAGEL 4 and antiSMASH 5.0 prediction. Annotation is based on CDD analysis. Figure S3. Multiple sequence alignment of roseocin family lanthipeptide precursor sequences to determine the conserved motif. Figure S4. Bayesian analysis of two LanMs of the same biosynthetic gene cluster from roseocin and lacticin 3147 families. Figure S5. (A) Distantly related BGCs of roseocin family from Walker et al. 2020 study, with multiple numbers of precursors in gene clusters. (B) Rosα homologs of these BGC are unusually short but contain the conserved motif S/TxxxxTxGCC. Figure S6. (A) Pairwise sequence alignment and percent identity of 42 LanM sequences (B) and the comparative cumulative frequency of identity percentage among leader, core, and LanM protein sequences of 42 BGCs. Figure S7. Sequence logo from multiple sequence alignment (A) Rosα and (B) Rosβ homologs from type 2 subclade showed highly conserved positions and possible sites of evolutionary variation. Figure S8. ESI-MS data showed that variants of Rosα were post-translationally modified by RosM, in E. coli BL21(DE3). Figure S9. Evaluation of antimicrobial activity in synergism with Rosβ after leader removal with proteinase K. Figure S10. Multiple sequence alignment of Rosα homologs to predict the ring topology in (A) Rosα and (B) Rosβ. Figure S11. Schematic representation of the primer design for site-directed mutagenesis. The arrowheads represent the site of mutation in the primer-primer overlapping region. Figure S12. A tanglegram between the 16S rRNA and the lanM gene tree indicates that intra-phyla horizontal gene transfer (HGT) is a major source of lanthipeptide diversity. Figure S13. Maximum likelihood phylogenetic tree of 16S rRNA from bacte [file 13568_2023_1536_MOESM1_ESM.docx]

**Phylogeny-Guided Genome Mining of Roseocin Family Lantibiotics to Generate Improved Variants of Roseocin**

Sandeep Chaudhary^#1^, Shweta Kishen^#1^, Mangal Singh^2^, Sunanda Jassal^1^, Reeva Pathania^1^, Kalpana Bisht^1^, Dipti Sareen*^1^

1. Department of Biochemistry, Panjab University, Chandigarh 160014, India
2. Department of Biosciences and Bioengineering, Indian Institute of Technology Roorkee, Roorkee 247667, India

**correspondence:* [*diptsare@pu.ac.in*](mailto:diptsare@pu.ac.in)

**Supplementary information:**

**Rosα variant generation by site-directed mutagenesis**

For single-site mutation, the PCR reactions were setup comprising of 50 μL contained a 5 ng template (pRSFDuet_RosM_Rosα), 2 µM primer pair, 200 µM dNTPs, and 3.75 Units of Pfu DNA polymerase**.** Initiated the PCR cycles at 95 °C for 5 minutes for the denaturation of template DNA, followed by 12 amplification cycles. Each amplification cycle consisted of 95 °C for 1 minute, annealing temperature (T*_a_*) was 5 °C less than T*_m no_* and was kept for 1 minute for the extension at 72 °C for 15 minutes (about 500 bp per minute for Pfu DNA polymerase). The PCR cycles were finished with an annealing step at 5 °C less than T*_m pp_* for 1 minute and a final extension step at 72 °C for 30 minutes (Liu and Naismith 2008). The PCR products obtained were treated with 5 units of DpnI at 37 ºC for one hour and the reaction was stopped by heating at 75 ºC for 15 minutes. The parental DNA template is eliminated by treating with DpnI, which digests the methylated and hemimethylated DNA template. Then 10 uL of each PCR reaction was analysed by agarose gel electrophoresis. After gel analysis, 50-120 ng of PCR product was transformed into chemically competent *E.coli* DH5α cells using the heat shock method, for nicks repair and plasmid amplification. After plasmid purification, the mutations were confirmed through gene sequencing. On confirmation, the mutation containing plasmid was transformed into the host *E.coli* BL21(DE3) for protein expression and *in vivo* post-translational modification by the plasmid bearing RosM (Singh et al. 2020).

**Purification of Roseocin alpha precursor peptide variants**

For purification of histidine tagged roseocin alpha precursor peptide variants, pRSFDuet containing mutated sequence for alpha precursor peptide variants were individually transformed in *E. coli* BL21(DE3) cells. Overnight culture of each respective recombinant was used to inoculate two litres of Luria Bertani broth containing kanamycin (40 µg/mL). The culture was then incubated at 37 °C, 180 rpm until the OD600 reached 0.6–0.8. At 18 ºC induction was done with 0.1 mM IPTG and kept at 180 rpm for another 24 hrs. The culture was harvested by centrifugation at 1500 g, 15min at 4 ºC and the cell pellet obtained was resuspended in 50mL start buffer (50 mM Tris-HCl, pH 8.0, 500 mM NaCl, 1 mM Imidazole, 1 mM PMSF). The cells were lysed via sonication using Sonics VC 505 sonicator. The lysate was cleared by centrifugation at 25,000 g for 30 min at 4 °C on SIGMA 3K30 centrifuge. The lysate was then loaded on 1 mL of Ni-sepharose high-performance (GE Healthcare) metal affinity resin (binding capacity- 40 mg/mL) in the presence of 1mM imidazole to reduce non-specific binding. After binding, the resin was washed with wash buffer 1 (start buffer containing 5 mM Imidazole and 8 M urea) & wash buffer 2 (washer buffer 1 containing 15 mM Imidazole) to remove non-specifically bound proteins and peptides. Finally, batch elution was done with elution buffer (washer buffer 1 with 500 mM Imidazole) and the peptide of interest was collected. Tricine SDS-PAGE gel was run to analyse and quantitate the peptide (Singh et al. 2020).

The IMAC purified alpha precursor peptide variants were further purified and desalted using RP-HPLC on Infinity1260 series HPLC system and semi-preparative column 300SB-C18 HPLC column (9.4 x 250 mm, 5 micron). The fractions containing variant peptide were lyophilised and peptide was cleaved to remove leader in order to obtain it’s bioactive form (just core region) using proteinase K (enzyme to substrate ratio 1:5). To obtain purified core peptide, RP-HPLC was done and fractions containing peptide were lyophilised. Quantification was also done using Nisin as standard via RP-HPLC for further antimicrobial analysis.

**ESI-MS Analysis:**

The ESI-MS analysis was done using Synapt XS HD Mass spectrometer (Waters Corporation, U.K.) for determining the mass of the roseocin alpha variant peptides before and after leader cleavage with GluC, as described earlier (Singh et al. 2020). The RP-HPLC purified samples of the full-length variants with leader region were dissolved in 80% ACN and 100 µL of their sample was loaded into the system for ESI-MS analysis. The cleaved or bioactive mutants were cleaned using Thermo Scientific™ Pierce™ C18 Spin Columns before ESI-MS. Data was recorded and analysed using the MassLynx 4.2 software from Waters.

**Agar Diffusion Assay**

Overnight 10 mL culture of *Micrococcus luteus* (ATCC 10240), grown in nutrient broth, was diluted to 0.1 OD_600_ in the fresh 5 mL nutrient broth. The 1 μL/mL of the culture was used for the inoculation in nutrient agar before plating. The culture was added in bearable to touch media. The nutrient agar culture plates were used for checking the activity of Rosα variant peptides and wild type as a positive control, in synergism with Rosβ in a 1:1 ratio.

Alpha variant precursor peptides were cleaved with proteinase K to obtain their bioactive form, as described earlier (Singh et al. 2020) and were spotted with Rosβ in nutrient agar culture plate, in a dose-dependent manner i.e 1 µg, 2 µg, 4 µg (each).

**FIGURES**

**Figure S1:** (A) Pie chart of the obtained hits, with most of the hits being from *actinobacteria* and *cyanobacteria* phylum. (B) Phylum-dependent clade formation of 100 RosM homologs in Bayesian analysis.

**Figure S2:** Complete BGCs of selected 42 LanMs based on BAGEL 4 and antiSMASH 5.0 prediction. Annotation is based on CDD analysis.

**Figure S3:** Multiple sequence alignment of roseocin family lanthipeptide precursor sequences to determine the conserved motif.

**Figure S4:** Bayesian analysis of two LanMs of the same biosynthetic gene cluster from roseocin and lacticin 3147 families.

**Figure S5:** (A) Distantly related BGCs of roseocin family from Walker et al. 2020 study, with multiple numbers of precursors in gene clusters. (B) Rosα homologs of these BGC are unusually short but contain the conserved motif S/TxxxxTxGCC.

**Figure S6:** (A) Pairwise sequence alignment and percent identity of 42 LanM sequences (B) and the comparative cumulative frequency of identity percentage among leader, core, and LanM protein sequences of 42 BGCs.

**Figure S7:** Sequence logo from multiple sequence alignment (A) Rosα and (B) Rosβ homologs from type 2 subclade showed highly conserved positions and possible sites of evolutionary variation.

**Figure S8:** ESI-MS data showed that variants of Rosα were post-translationally modified by RosM, in *E. coli* BL21(DE3)*.*

**Figure S9:** Evaluation of antimicrobial activity in synergism with Rosβ after leader removal with proteinase K.

**Figure S10:** Multiple sequence alignment of Rosα homologs to predict the ring topology in (A) Rosα and (B) Rosβ.

**Figure S11:** Schematic representation of the primer design for site-directed mutagenesis. The arrowheads represent the site of mutation in the primer-primer overlapping region.

**Figure S12:** A tanglegram between the 16S rRNA and the *lanM* gene tree indicates that intra-phyla horizontal gene transfer (HGT) is a major source of lanthipeptide diversity.

**Figure S13:** Maximum likelihood phylogenetic tree of 16S rRNA from bacterial species of (A) *actinobacteria* and (B) *cyanobacteria*, in correlation of their BGCs organization.

**Figure S14:** (A, B) Tanglegram between the phylogenetic tree of LanM and their associated lanthipeptide leader/core in Dendroscope.

**TABLES**

**Table S1:** 42 selected hits from RosM search in the NCBI database.

**Table S2:** A comparison of the roseocin and lacticin 3147 families of two LanM-two precursor genes.

**Table S3:** Accession number of ProcM family LanMs from Cubillos-Ruiz et al. 2017.

**Table S4:** Calculated (by Expasy) and observed (by ESI-MS) average mass of full length (with leader region) post-translationally modified Rosα-wild type and its variants. Selected positions for mutant generation are bold and italicized.

**Table S5:** Calculated (by Expasy) and observed (by MALDI-TOF MS) monoisotopic mass of Rosα variants after leader cleavage using endoproteinase GluC (leaving an overhang of four amino acid residues) reduced with TCEP only & TCEP reduced peptides alkylated with IAA.

**Table S6:** b and y ions obtained in MS-MS fragmentation of Rosα variants.

**Table S7:** The list of primers used for the SDM-PCR in the generation of variants (F-forward and R-reverse primer).

**Figure S1: (A) Pie chart of the obtained hits, with most of the hits being from *actinobacteria* and *cyanobacteria* phylum. (B) Phylum-dependent clade formation of 100 RosM homologs in Bayesian analysis.** Lack of any hit from firmicutes seems to be as a result of independent evolution of roseocin from the lacticin 3147 family.

**A.**

**
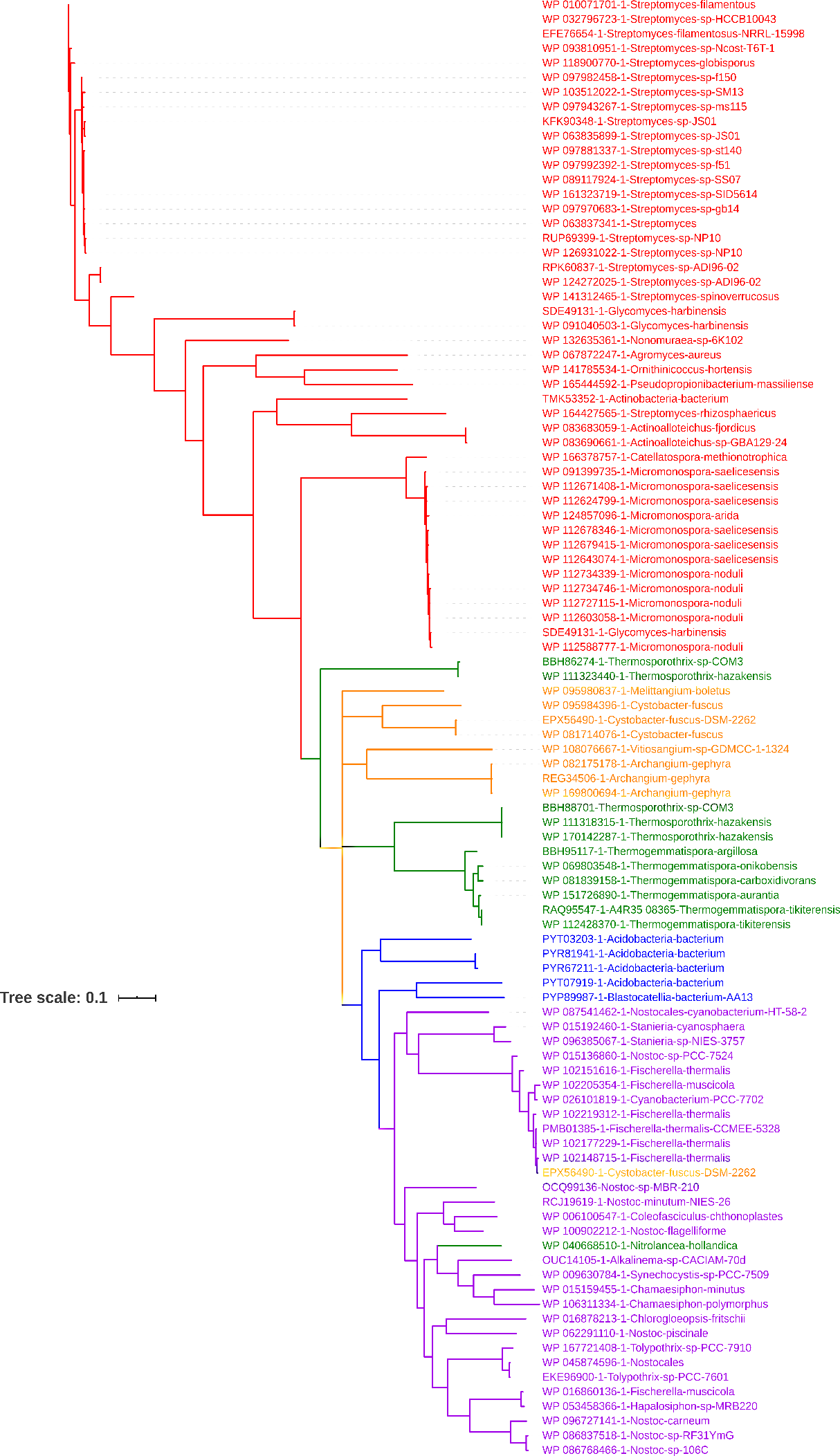
B.**

**Figure S2: Complete BGCs of selected 42 LanMs based on BAGEL 4 and antiSMASH 5.0 prediction. Annotation is based on CDD analysis.**

**Figure S3: Multiple sequence alignment of roseocin family lanthipeptide precursor sequences to determine the conserved motif.** (A) Rosα homologs contain S/TxxxxTxGCC at N-terminal and (B) Rosβ homologs contain GS/TxxxxS/TxGCC motif at C-terminal. Additional members were found in the Walker et al. 2020 study (marked with #). Additional members are of the type II BGC gene cluster (single LanM two precursor). Dehydratable Ser/Thr are highlighted with red colour, Cys residues involved in lanthionine/methyl-lanthionine rings are highlighted with cyan colour while Cys residue involved in disulphide linkage are highlighted with green colour.

**Figure S4: Bayesian analysis of two LanMs of the same biosynthetic gene cluster from roseocin and lacticin 3147 families.** Roseocin and lacticin 3147 families have evolved differently, as their clades are well separated.

**Figure S5: (A) Distantly related BGCs of roseocin family from Walker et al. 2020 study, with the multiple numbers of precursors in gene clusters.** Using *S. alni* LanM from Walker et al. 2020 as a query, *Hamadaea* sp. BGC was identified by us from NCBI database and possibly more such clusters could be identified in the future. **(B) Rosα homologs of these BGCs are unusually short but do contain the conserved motif S/TxxxxTxGCC.** LanA: precursor peptide; LanM: modification enzyme; LanC domain: cyclase domain; HP: Hypothetical Protein; LanT: dual function peptidase-domain containing transporter.

**Figure S6: (A) Pairwise sequence alignment and percent identity of 42 LanM sequences (B) and the comparative cumulative frequency of identity percentage among leader, core, and LanM protein sequences of 42 BGCs.** LanM pairwise sequence identity displayed a trend more like leader sequence, despite high sequence variability. This suggested a correlation and provided another reason to test the coevolution hypothesis.

**Figure S7: Sequence logo from multiple sequence alignment (A) Rosα and (B) Rosβ homologs from type 2 subclade showed highly conserved positions and possible sites of evolutionary variation.** Marked positions (with rectangle) are amino acids position of relevance due to their semi-conserved nature of the position (i.e., variation in not more than four amino acids) and at the same time substitutions could have larger effect on roseocin’s antimicrobial activity. Numbering shown below the weblogo has resulted from multiple sequence alignment (Figure S3) and hence, does not correspond to the actual amino acid positions of Rosα and Rosβ.

**Figure S8: ESI-MS data showed that variants of Rosα were successfully post-translationally modified by RosM, in *E. coli* BL21(DE3)*.*** Observed mass peaks, corresponding to [M-4H_2_O-2H+2H^+^]^2+^ ion species along with an additional peak for the α-N-gluconoylated product as +89 Da adduct (*), in all the heterologously produced Rosα variants were found to be in agreement with four fold dehydrated species. Mass reduction of 2 Da (-2H)  is as a  result of one disulphide bond formed between two sulfhydryl residues (Cys13 and Cys33) (inset). The colour codes used are: wild-type Rosα - black, variant L8F - green, variant L8W - red, variant S12W- yellow and variant V22Q - cyan. Final leader removal sites are shown in inset. Enodproteinase GluC digestion before tandem MS and proteinase K digestion (to obtain core peptides only) before antimicrobial activity determination was done. Table S4 represents the calculated and observed mass of the respective species.

**Figure S9: Evaluation of antimicrobial activity in synergism with Rosβ after leader removal with proteinase K.** Synergistic antimicrobial activity was tested at three different concentrations (4 μg, 2 μg and 1 μg, top to bottom) with an equal amount of Rosβ. All variants are bioactive and the zone of inhibition has improved significantly in the case of Rosα L8F as compared to wild type Rosα.

**Figure S10: Multiple sequence alignment of homologs to predict the ring topology** **in (A) Rosα and (B) Rosβ.** Using simultaneous deletion of both of the Ser/Thr and Cys residues pair in homologs as the hallmark, the ring pattern was decoded in overlapping region (solid lines) of roseocin peptides. Disulphide bond in Rosα (shown with solid green colour); non-overlapping rings (ring A and B) in Rosβ have been already determined on the basis of tandem MS (Singh et al. 2020). Essential motifs amino acids in roseocin peptide (shown with dotted lines) have not shown any substitution and hence is still speculative.

**Figure S11: Schematic representation of the primer design for site-directed mutagenesis. The arrowheads represent the site of mutation in the primer-primer overlapping region.**

**
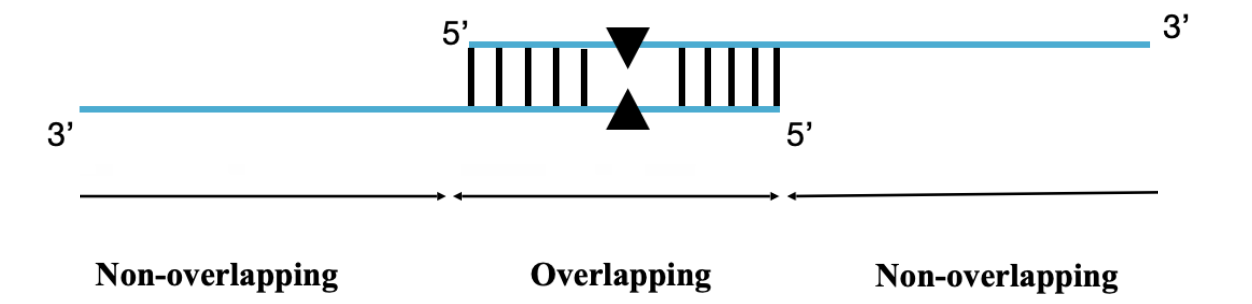
**

**Figure S12: A tanglegram between the 16S rRNA and the *lanM* gene tree indicates that intra-phyla horizontal gene transfer (HGT) is a major source of lanthipeptide diversity.** The 16S rRNA data were available for only 37 microorganisms, out of 42 hits, in the SILVA reference database.

**Figure S13: Maximum likelihood phylogenetic tree of 16S rRNA from bacterial species of (A) *actinobacteria* and (B) *cyanobacteria*, in correlation of their BGCs organization.** Value from 500 replicates bootstrap test is indicated on each branch. Type 1-3 BGC organization are based on earlier analysis from LanM based phylogenetic tree (Figure 2 and 3).

**Figure S14: (A, B) Tanglegram between the phylogenetic tree of LanM and their associated lanthipeptide leader/core in Dendroscope.** A linear relation between cognate lanthipeptide leader and LanM suggests a history of coevolution between both, *vis-à-vis* independently evolving lanthipeptide core sequences. As a representative, Rosα precursor sequences have been used here.

**Table S1: 42 selected hits from RosM search in the NCBI database.**

| Organism | LanM Accesion | Reference genome | Precursors |
| --- | --- | --- | --- |
| *Streptomyces rhizosphaericus* | WP_164427565.1 | NZ_JAAIKT010000015.1 | 3 |
| *Actinoalloteichus fjordicus* strain ADI127 | WP_083683059.1 | NZ_CP016076.1 | 3 |
| *Streptomyces filamentosus* NRRL 11379 | WP_010071701.1 | NZ_ABYX02000001.1 | 2 |
| *Streptomyces* sp. ADI96-02 | WP_124272025.1 | NZ_ML123034.1 | 2 |
| *Streptomyces spinoverrucosus* NBRC 14228 | WP_141312465.1 | NZ_BJND01000044.1 | 2 |
| *Glycomyces harbinensis* CGMCC 4.3516 | WP_091040503.1 | NZ_FNAD01000024.1 | 2 |
| *Nonomuraea* sp. 6K102 | WP_132635361.1 | NZ_SMLD01000099.1 | 2 |
| *Agromyces aureus* strain AR33 | WP_067872247.1 | NZ_CP013979.1 | 2 |
| *Ornithinicoccus hortensis* strain DSM 12335 | WP_141785534.1 | NZ_VFOP01000001.1 | 2 |
| *Pseudopropionibacterium massiliense* Marseille-P6184 | WP_165444592.1 | NZ_LR027842.1 | 2 |
| *Catellatospora methionotrophica* strain IMSNU 22006 | WP_166378757.1 | NZ_JAAOTJ010000001.1 | 2 |
| *Micromonospora arida* strain LB32 | WP_124857096.1 | NZ_QGSY01000175.1 | 2 |
| *Micromonospora noduli* strain MED15 | WP_112734339.1 | NZ_PYAC01000001.1 | 2 |
| *Vitiosangium* sp. GDMCC 1.1324 | WP_108076667.1 | NZ_PZOX01000029.1 | 1 |
| *Thermogemmatispora carboxidivorans* strain PM5 | WP_081839158.1 | NZ_JNIM01000001.1 | 1 |
| *Thermosporothrix hazakensis* strain SK20-1 | WP_170142287.1 | NZ_BIFX01000001.1 | 1 |
| *Cystobacter fuscus* strain DSM 52655 | WP_095984396.1 | NZ_CP022098.1 | 1 |
| *Cystobacter fuscus* DSM 2262 | WP_081714076.1 | NZ_ANAH02000066.1 | 1 |
| *Archangium gephyra* strain DSM 2261 | WP_082175178.1 | NZ_QUMU01000003.1 | 1 |
| *Melittangium boletus* DSM 14713 | WP_095980837.1 | NZ_CP022163.1 | 1 |
| *Acidobacteria bacterium* isolate gp4 AA17 | PYT03203.1 | QHXP01000290.1 | 1 |
| *Nostocales cyanobacterium* HT-58-2 | WP_087541462.1 | NZ_CP019636.1 | 2 |
| *Stanieria cyanosphaera* PCC 7437 | WP_015192460.1 | NC_019748.1 | 4 |
| *Fischerella muscicola* CCMEE 5323 | WP_102205354.1 | NZ_NRQW01000326.1 | 1 |
| *Fischerella thermalis* CCMEE 5201 | WP_102151616.1 | NZ_NMQK01000460.1 | 1 |
| *Nostoc* sp. PCC 7524 | WP_015136860.1 | NC_019684.1 | 1 |
| *Nostoc sp.* MBR 210 | OCQ99136.1 | MBRD01000004.1 | 1 |
| *Acidobacteria bacterium* isolate gp4 AA12 | PYT07919.1 | QHXT01000195.1 | 2 |
| *Blastocatellia* bacterium AA13 | PYP89987.1 | QHVH01000002.1 | 1 (2 distant) |
| *Nitrolancea hollandica* | WP_040668510.1 | NZ_CAGS01000450.1 | 1 |
| *Alkalinema* sp. CACIAM 70d | OUC14105.1 | MUGG01000119.1 | 2 |
| *Chamaesiphon minutus* PCC 6605 | WP_015159455.1 | NC_019697.1 | 3 (4 distant) |
| *Synechocystis sp.* PCC 7509 | WP_009630784.1 | NZ_ALVU02000002.1 | 9 |
| *Nostoc piscinale* CENA21 | WP_062291110.1 | NZ_CP012036.1 | 1 |
| *Chlorogloeopsis fritschii* PCC 6912 | WP_016878213.1 | NZ_AJLN01000088.1 | 1 |
| *Coleofasciculus chthonoplastes* PCC 7420 | WP_006100547.1 | NZ_DS989847.1 | 1 |
| *Nostoc minutum* NIES-26 | RCJ19619.1 | LXQD01000339.1 | 1 |
| *Tolypothrix sp.* PCC 7910 | WP_167721408.1 | NZ_CP050440.1 | 1 |
| *Tolypothrix sp.* PCC 7601 | EKE96900.1 | NZ_JH930378.1 | 1 |
| *Hapalosiphon* sp. MRB220 | WP_053458366.1 | NZ_LIRN01000084.1 | 1 |
| *Nostoc carneum* NIES-2107 | WP_096727141.1 | NZ_AP018180.1 | 1 |
| *Nostoc* sp. 106C | WP_086768466.1 | NZ_MTAW01000225.1 | 1 |

**Table S2: A comparison of the roseocin family and lacticin 3147 families of two LanM-two precursor genes.** LanMs of the roseocin family shares a high sequence identity of 50%, which is an indicator of gene duplication. However, lacticin 3147 family members contain LanMs and the precursors’ leader region with much lower identity, indicating distant divergent evolution of leader-LanM dual in them. A similar change in leader sequences and LanMs corroborate to the leader and LanM coevolution.

| Lantibiotic/ Lantibiotic Producer | Precursor / LanMs | Identity among LanMs (%) | Identity among leader sequences (%) | Reference |
| --- | --- | --- | --- | --- |
| Roseocin family | | | | |
| Roseocin | 2/1 | --- | 47.5 | (Singh et al. 2020) |
| *C. methionotrophica* | 2/2 | 49.21 | 52.38 | This work |
| *M. arida* | 2/2 | 50.37 | 57.14 | This work |
| *M. noduli* | 2/2 | 50.00 | 54.76 | This work |
| Lacticin 3147 family | | | | |
| Lacticin 3147 | 2/2 | 24.27 | 31.03 | (Ryan et al. 1996) |
| Lichenicdin | 2/2 | 29.27 | 37.5 | (Begley et al. 2009) |
| Haloduracin | 2/2 | 27.42 | 22.86 | (Mcclerren et al. 2006) |
| Thusin | 3/2 | 25.16 | 31.03  (Between LanA1 and LanA2/A2’)  93.33*  (Between LanA2 and LanA2’) | (Xin et al. 2016) |
| Bht | 2/2 | 24.87 | 18.52 | (Hyink et al. 2005) |
| Smb | 2/2 | 24.74 | 19.23 | (Yonezawa and Kuramitsu 2005) |

*Two 93.33% identical precursors.

**Table S3: Accession number of ProcM family LanMs from Cubillos-Ruiz et al. 2017.**

| Organism | LanM Accesion Id | Reference genome | Start-Stop |
| --- | --- | --- | --- |
| *Synechococcus* MIT S9508 | WP_156485153.1 | NZ_LVHU01000004.1 | c65416-62033 |
| *Synechococcus* MIT S9504 | WP_066911479.1 | [NZ_LVHT01000022.1](https://www.ncbi.nlm.nih.gov/nuccore/NZ_LVHT01000022.1) | 25824-29207 |
| *Synechococcus* RS9916 | WP_007098833.1 | NZ_DS022299.1 | 1460676-1463852 |
| *Synechococcus* KORDI-100 | WP_051847417.1 | NZ_CP006269.1 | c1799507-1796166 |
| *Prochlorococcus* MIT 0701 | WP_152562293.1 | NZ_JNBA01000007.1 | 4354-7584 |
| *Prochlorococcus marinus* MIT 9313 | WP_011129629.1 | NC_005071.1 | c283341-280135 |
| *Prochlorococcus marinus* MIT 9303 | ABM78842.1 | CP000554.1 | 1865532-1868738 |
| *Prochlorococcus marinus* MIT 1327 | KZR79196.1 | LVHR01000034.1 | 434491-437715 |
| *c means complementary region. | | | |

**Table S4: Calculated (by Expasy) and observed (by ESI-MS) average mass of full length (with leader region) post-translationally modified Rosα-wild type and its variants.** Selected positions for mutant generation are bold and coloured red.

**GSSHHHHHHSQDP**MDIVRSWKDADYRLSLGSEAPAHPSGEGLTAITDEELTEINGAGSGVLGT**L**GCC**S**CLPWYSGWT**V**CGLACNPGKPCKN

| **Peptide** | **Cal. m/z**  **[M-4H_2_O-2H+2H^+^]^+2^** | **Obs. m/z**  **[M-4H_2_O-2H+2H^+^]^+2^** | **Difference** | **Error** |
| --- | --- | --- | --- | --- |
| **WT** | 4770.8208 | 4768.5166 | -2.3042 | -0.000482 |
| **L8W** | 4806.9995 | 4804.6836 | -2.3139 | -0.000481 |
| **L8F** | 4787.8158 | 4785.1709 | -2.1450 | -0.000448 |
| **S12W** | 4820.3458 | 4817.1875 | -3.1568 | -0.000654 |
| **V22Q** | 4785.3158 | 4782.6694 | -2.6469 | -0.000553 |

**Table S5: Calculated (by Expasy) and observed (by MALDI-TOF MS) monoisotopic mass of Rosα variants after leader cleavage using endoproteinase GluC (leaving an overhang of four amino acid residues) reduced with TCEP only & TCEP reduced peptides alkylated with IAA.** Sites of substitution are bold and coloured red.

INGAGSGVLGT**L**GCC**S**CLPWYSGWT**V**CGLACNPGKPCKN

|  | **After reduction with TCEP** | | | **After TCEP+IAA** | | |
| --- | --- | --- | --- | --- | --- | --- |
| **Peptide** | **Cal. m/z**  **[M-4H_2_O+H]^+^** | **Obs. m/z**  **[M-4H_2_O+H]^+^** | **Error** | **Cal. m/z**  **[M-4H_2_O-2H+2IAA+H]^+^** | **Obs. m/z**  **[M-4H_2_O-2H+2IAA+H]^+^** | **Error** |
| **WT** | 3814.76 | 3815.12 | -0.000094 | 3926.73 | 3928.79 | -0.000525 |
| **L8W** | 3887.75 | 3887.79 | -0.000010 | 3999.75 | 4001.70 | -0.000488 |
| **L8F** | 3848.76 | 3848.62 | 0.000036 | 3960.76 | 3963.25 | -0.000629 |
| **S12W** | 3913.81 | 3913.96 | -0.000038 | 4025.81 | 4027.64 | -0.000455 |
| **V22Q** | 3843.75 | 3843.52 | 0.000060 | 3955.75 | 3957.51 | -0.000445 |

**Table S6: b and y ions obtained in MS-MS fragmentation of Rosα variants.** Corresponding raw data files are provided in Mendeley dataset. Sites of mutagenesis are bold and coloured red.

-1 +1 8 12 22 +35

INGAGSGVLGT**L**GCC**S**CLPWYSGWT**V**CGLACNPGKPCKN

**L8F**

| Ion | Calc. m/z | Obs. m/z | Sequence | Diff. | Error |
| --- | --- | --- | --- | --- | --- |
| y4 | 518.6092 | 518.1012 | PCKN **[1xCarbamidomethyl]** | -0.508 | -0.00098 |
| y5 | 646.7818 | 646.1544 | KPCKN **[1xCarbamidomethyl]** | -0.627 | -0.00097 |
| y7 | 800.9486 | 800.2066 | PGKPCKN **[1xCarbamidomethyl]** | -0.742 | -0.00093 |
| y8 | 915.0515 | 914.2312 | NPGKPCKN **[1xCarbamidomethyl]** | -0.820 | -0.0009 |
| y17 | 1788.109 | 1786.465 | GWTVCGLACNPGKPCKN **[1xCarbamidomethyl; 1xDehydrated]** | -1.644 | -0.00092 |
| y18 | 1857.171 | 1855.484 | SGWTVCGLACNPGKPCKN **[1xCarbamidomethyl; 2xDehydrated]** | -1.687 | -0.00091 |
| y19 | 2020.345 | 2018.512 | YSGWTVCGLACNPGKPCKN **[1xCarbamidomethyl; 2xDehydrated]** | -1.833 | -0.00091 |
| b21 | 2109.437 | 2108.568 | INGAGSGVLGTFGCCSCLPWY **[1xCarbamidomethyl; 2xDehydrated]** | -0.868 | -0.00041 |
| y21 | 2303.671 | 2301.622 | PWYSGWTVCGLACNPGKPCKN **[1xCarbamidomethyl; 2xDehydrated]** | -2.049 | -0.00089 |
| y22 | 2416.829 | 2415.596 | LPWYSGWTVCGLACNPGKPCKN **[1xCarbamidomethyl; 2xDehydrated]** | -1.232 | -0.00051 |
| y37 | 3738.34 | 3736.576 | GAGSGVLGT**F**GCCSCLPWYSGWTVCGLACNPGKPCKN **[2xCarbamidomethyl; 4xDehydrated]** | -1.763 | -0.00047 |
| y38 | 3852.443 | 3849.817 | NGAGSGVLGT**F**GCCSCLPWYSGWTVCGLACNPGKPCKN **[2xCarbamidomethyl; 4xDehydrated]** | -2.625 | -0.00068 |

**L8W**

| Ion | Calc. m/z | Obs. m/z | Sequence | Diff. | Error |
| --- | --- | --- | --- | --- | --- |
| y7 | 800.9486 | 801.226 | PGKPCKN **[1xCarbamidomethyl]** | 0.277 | 0.000346 |
| y8 | 915.0515 | 914.1805 | NPGKPCKN **[1xCarbamidomethyl]** | -0.871 | -0.00095 |
| y18 | 1857.171 | 1855.5 | SGWTVCGLACNPGKPCKN **[1xCarbamidomethyl; 2xDehydrated]** | -1.671 | -0.00090 |
| y19 | 2020.345 | 2019.498 | YSGWTVCGLACNPGKPCKN **[1xCarbamidomethyl; 2xDehydrated]** | -0.847 | -0.00042 |
| y21 | 2303.671 | 2302.562 | PWYSGWTVCGLACNPGKPCKN **[1xCarbamidomethyl; 2xDehydrated]** | -1.109 | -0.00048 |
| y37 | 3777.376 | 3774.514 | GAGSGVLGT**W**GCCSCLPWYSGWTVCGLACNPGKPCKN **[2xCarbamidomethyl; 4xDehydrated]** | -2.861 | -0.00076 |

**S12W**

| Ion | Calc. m/z | Obs. m/z | Sequence | Diff. | Error |
| --- | --- | --- | --- | --- | --- |
| y7 | 800.9486 | 800.1639 | PGKPCKN **[1xCarbamidomethyl]** | -0.7847 | -0.00098 |
| y8 | 915.0515 | 914.1999 | NPGKPCKN **[1xCarbamidomethyl]** | -0.851 | -0.00093 |
| b17 | 1614.896 | 1613.249 | INGAGSGVLGTLGCCWC **[1xCarbamidomethyl; 2xDehydrated]** | -1.647 | -0.00102 |
| b18 | 1728.054 | 1726.521 | INGAGSGVLGTLGCCWCL **[1xCarbamidomethyl; 2xDehydrated]** | -1.532 | -0.00089 |
| y18 | 1857.171 | 1855.331 | SGWTVCGLACNPGKPCKN **[1xCarbamidomethyl; 2xDehydrated]** | -1.840 | -0.00099 |
| y19 | 2020.54 | 2020.64 | YSGWTVCGLACNPGKPCKN **[1xCarbamidomethyl; 2xDehydrated]** | 0.099 | 4.93E-05 |
| y21 | 2303.671 | 2301.487 | PWYSGWTVCGLACNPGKPCKN **[1xCarbamidomethyl; 2xDehydrated]** | -2.183 | -0.00095 |
| y37 | 3803.456 | 3801.349 | GAGSGVLGTLGCC**W**CLPWYSGWTVCGLACNPGKPCKN **[2xCarbamidomethyl; 4xDehydrated**] | -2.106 | -0.00055 |
| y38 | 3917.559 | 3918.062 | NGAGSGVLGTLGCC**W**CLPWYSGWTVCGLACNPGKPCKN **[2xCarbamidomethyl; 4xDehydrated]** | 0.502 | 0.00012 |

**V22Q**

| Ion | Calc. m/z | Obs. m/z | Sequence | Diff. | Error |
| --- | --- | --- | --- | --- | --- |
| y7 | 800.9486 | 800.1941 | PGKPCKN **[1xCarbamidomethyl]** | -0.754 | -0.00094 |
| y8 | 915.0515 | 914.1971 | NPGKPCKN **[1xCarbamidomethyl]** | -0.854 | -0.00093 |
| b18 | 1628.921 | 1629.3156 | INGAGSGVLGTLGCCSCL **[1xCarbamidomethyl; 2xDehydrated]** | 0.394 | 0.000242 |
| y18 | 1886.17 | 1885.4013 | SGWT**Q**CGLACNPGKPCKN **[1xCarbamidomethyl; 2xDehydrated]** | -0.768 | -0.00041 |
| y19 | 2049.343 | 2047.4187 | YSGWT**Q**CGLACNPGKPCKN **[1xCarbamidomethyl; 2xDehydrated]** | -1.924 | -0.00094 |
| y21 | 2332.669 | 2330.4814 | PWYSGWT**Q**CGLACNPGKPCKN **[1xCarbamidomethyl; 2xDehydrated]** | -2.187 | -0.00094 |
| y37 | 3733.322 | 3732.1557 | GAGSGVLGTLGCCSCLPWYSGWT**Q**CGLACNPGKPCKN **[2xCarbamidomethyl; 4xDehydrated]** | -1.165 | -0.00031 |
| y38 | 3847.424 | 3846.3326 | NGAGSGVLGTLGCCSCLPWYSGWT**Q**CGLACNPGKPCKN **[2xCarbamidomethyl; 4xDehydrated]** | -1.091 | -0.00028 |

**Table S7: The list of primers used for the SDM-PCR in the generation of variants (F-forward and R-reverse primer).** The T*_m pp_* is the primer-primer overlapping region’s (shown in bold and italics) melting temperature. T*_m no_* is non-overlapping primer region’s (shown in normal font times new roman) melting temperature. T*_a_* is the annealing temperature. The small alphabets in the sequences show the site of mutation.

| **Primers** | **Sequences** | **T*_m pp_* (°C)** | **T*_m no_*(°C)** | **T*_a pp_* (°C)** | **T*_a no_* (°C)** |
| --- | --- | --- | --- | --- | --- |
| RosA2 L8F/**F** | 5’***CACTttcGGGTGC***TGCTCGTGCCTGCCG3’ | 46.1 | 59.3 | 41.1 | 54.3 |
| RosA2 L8F/**R** | 5’***GCACCCgaaAGTG***CCGAGAACGCCGGAGCC3’ | 46.1 | 61.9 | 41.1 | 56.9 |
| RosA2 L8W/**F** | 5’***CACTtggGGGTGC***TGCTCGTGCCTGCCGTG3’ | 49.3 | 61.8 | 44.3 | 56.8 |
| RosA2 L8W/**R** | 5’***GCACCCccaAGTG***CCGAGAACGCCGGAGCC3**’** | 49.3 | 61.9 | 44.3 | 56.9 |
| RosA2 S12W/**F** | 5’***TGCTGCtggTGCCT***GCCGTGGTATTCGGGTTGGACC3’ | 48.2 | 62.6 | 43.2 | 57.6 |
| RosA2 S12W/**R** | 5’***AGGCAccaGCAGCA***CCCGAGAGTGCCGAGAACGC3’ | 54.4 | 63 | 49.4 | 58 |
| RosA2 V22Q/**F** | 5’***TGGACCcaaTGCGGT***CTCGCCTGCAACCCCGGTAAGC3’ | 54.1 | 65 | 49.1 | 60 |
| RosA2 V22Q/**R** | 5’***ACCGCAttgGGTCCA***ACCCGAATACCACGGCAGGCAC3’ | 54.1 | 64.2 | 49.1 | 59.2 |

**References:**

Begley M, Cotter PD, Hill C, Ross RP (2009) Identification of a novel two-peptide lantibiotic, lichenicidin, following rational genome mining for LanM proteins. Appl Environ Microbiol 75:5451–5460. https://doi.org/10.1128/AEM.00730-09

Cubillos-Ruiz A, Berta-Thompson JW, Becker JW, Van Der Donk WA, Chisholm SW (2017) Evolutionary radiation of lanthipeptides in marine cyanobacteria. Proc Natl Acad Sci USA 114:E5424–E5433. https://doi.org/10.1073/pnas.1700990114

Hyink O, Balakrishnan M, Tagg JR (2005) *Streptococcus rattus* strain BHT produces both a class I two-component lantibiotic and a class II bacteriocin. FEMS Microbiol Lett 252:235–241. https://doi.org/10.1016/j.femsle.2005.09.003

Liu H, Naismith JH (2008) An efficient one-step site-directed deletion, insertion, single and multiple-site plasmid mutagenesis protocol. BMC Biotechnol 8:91. https://doi.org/10.1186/1472-6750-8-91

Mcclerren AL, Cooper LE, Quan C, Thomas PM, Kelleher NL, Donk WA Van Der (2006) Discovery and in vitro biosynthesis of haloduracin, a two-component lantibiotic. Proc Natl Acad Sci USA 103:17243–17248

Ryan MP, Rea MC, Hill C, Ross RP (1996) An application in cheddar cheese manufacture for a strain of *Lactococcus lactis* producing a novel broad-spectrum bacteriocin, lacticin 3147. Appl Environ Microbiol 62:612–619

Singh M, Chaudhary S, Sareen D (2020) Roseocin, a novel two‐component lantibiotic from an actinomycete. Mol Microbiol 113:326–337. https://doi.org/10.1111/mmi.14419

Walker MC, Eslami SM, Hetrick KJ, Ackenhusen SE, Mitchell DA, van der Donk WA (2020) Precursor peptide-targeted mining of more than one hundred thousand genomes expands the lanthipeptide natural product family. BMC Genomics 21:387. https://doi.org/10.1186/s12864-020-06785-7

Xin B, Zheng J, Liu H, Li J, Ruan L, Peng D (2016) Thusin, a novel two-component lantibiotic with potent antimicrobial activity against several Gram-positive pathogens. 7:1-12. https://doi.org/10.3389/fmicb.2016.01115

Yonezawa H, Kuramitsu HK (2005) Genetic analysis of a unique bacteriocin, Smb, produced by *Streptococcus mutans* GS5. Antimicrob Agents Chemother 49:541–548. https://doi.org/10.1128/AAC.49.2.541-548.2005
