# Supplementary material for: Phylogeny-guided genome mining of roseocin family lantibiotics to generate improved variants of roseocin
Source: AMB Express. 2023 Mar 20;13:34. doi: 10.1186/s13568-023-01536-9 (PMC10027976; doi:10.1186/s13568-023-01536-9)
Supplement: Supplementary file 2 — Additional file 2. Amino acid sequence of lanthipeptide precursors associated with (A) 42 BGCs of Figure 2 (main text), and (B) additional roseocin homologs from Walker et al, 2020 study. [file 13568_2023_1536_MOESM2_ESM.docx]

**Phylogeny-Guided Genome Mining of Roseocin Family Lantibiotics to Generate Improved Variants of Roseocin**

Sandeep Chaudhary^#1^, Shweta Kishen^#1^, Mangal Singh^2^, Sunanda Jassal^1^, Reeva Pathania^1^, Kalpana Bisht^1^, Dipti Sareen*^1^

1. Department of Biochemistry, Panjab University, Chandigarh 160014, India
2. Department of Biosciences and Bioengineering, Indian Institute of Technology Roorkee, Roorkee 247667, India

**correspondence:* [*diptsare@pu.ac.in*](mailto:diptsare@pu.ac.in)

**Amino acid sequence of lanthipeptide precursors associated with (A) 42 BGCs of Figure 2 (main text), and (B) additional roseocin homologs from Walker et al, 2020 study.** Underlined region denotes lanthipeptide leader sequence. Precursor peptide sequences in red colour are the N11P family members**.** Associated LanM sequence id from Walker et al, 2020 study is given in parenthesis (with multiple id mentioned for redundant hits in different bacterial genomes). Precursor peptides without accession number have been identified manually in the current study by ORFfinder.

**A.**

**Actinobacteria**

>Streptomyces rhizosphaericus WP_164427567.1_Lan A1

MDIVRAWKDPEYRDTLAAAPDHPSGSHGLARLEMSELASAAGAGTESTLTVGCCASPKTFTLTSPIFCTI

TLSICP

>Streptomyces rhizosphaericus WP_164427571.1_Lan A2A

MDTVRAWKDPEYRRSLPCPPQHPSGVPGLSMINPSDLETPAGGVGSGTICLTKTFTLVSPVYCVITLAVC

>Streptomyces rhizosphaericus WP_164427569.1_Lan A2B

MDAVKAWKDPEYRNSLSAAERAELPDNPAGLVSLSHKDLTDVSGAYTTWLCASVAVSAWLCFGTKGYQSI

GCC

>Actinoalloteichus fjordicus WP_075739929.1_Lan A1

MDIVRSWKDPEFRASFPSAAHPAGPAELTPLELGDASQAAGGRTENLATFGCCFSPQTFTYTSPILCSVT

LSVC

>Actinoalloteichus fjordicus WP_075739931.1_Lan A2A

MDVVRSWKDPEYRASHGEFTAHPSGPARLSRLELGEIEAAGAGSARICGPTATLTLTLPAWCIVSLSLC

>Actinoalloteichus fjordicus WP_075739930.1_Lan A2B

MSTIRAWKDPEYRDSLAKDALAGGNAHPAGPSLLTSSELEMAQGGTTWPCFGITVSIAACQGTKGYGSVG

CC

>Agromyces aureus WP_067881095.1_Lan A1

MSLVDSWKDADRGRSVHHPSGVVDLAELDELEMDDAAGVGTNPLGTFGCCWCVPWWSGFTVCGAVCGTVC

KW

>Agromyces aureus ORF2_Lan A2

MTRAVLEWREPLVREADATHPSGIVELSDSDMEMLDGGTGWA**C**ATVSIGVGISLAW**C**SPGGTF**C**GS**C**TFGTSG**CC**R

>Ornithinicoccus hortensis WP_141785536.1_Lan A1

MDIVRSWKDPQSRSADMAHPSGSAEIAELTELEMDEVSGAGSGLVGTFGCCWCVPWYSGWTVCGLVCDHP

GC

>Ornithinicoccus hortensis WP_141785537.1_Lan A2

MTTTTAWKDPQARGSDLDIHPAGVVELSDDDMDMVAGGTGWACAIVTATIAITVTKCSPDGTLCGSCQMG

TRGCC

>Pseudopropionibacterium massiliense WP_130873166.1_Lan A1

MRNDDVNMNSSEGLLAGVDHPSGLIDLTELSDIELDETSGAAGTGLVGTFGCCWCVPWYSGWTVCGLVCS

HPGCKR

>Pseudopropionibacterium massiliense WP_130873165.1_Lan A2

MNSSDSAILLHNPAGIVELDTDDMDLDMTHGGTTWWCAIATATIALTATKCSPDGTLCGSCQMGTRGCC

>Nonomuraea sp. 6K102 WP_132635292.1_Lan A1

MDTVRAWKDPVYRRSLGASAPSHPAGQPTVTVLSDAELVDISGAGTRHHGTTGCCSCNPAWSAAWGIWCNPPSGC

>Nonomuraea sp. 6K102 WP_132635289.1_Lan A2

MDVLRAWKDPVYRSGLDQAKLPSHPAGVVDLTDDQLNSVDGGTTTWLCATAGVVTATATVCSPSGTLCGSCSHGSSGCC

>Glycomyces harbinensis SDE49113.1_Lan A1

MDIVRSWKEPEYRRSLGDDAPEHPAGTELTEVSELSLTSVGGAAGTQHMGTFGCCWCLPWYSGWTVCGSL

CSPKYVCD

>Glycomyces harbinensis SDE49091.1_Lan A2

MKTTRAWKDPEYRRELGEDAQNNPAGLVDLNDDAVGSIAGGSTWGCVTATIALTVTVCSPTGTLCGSCQM

GTRGCC

> Streptomyces spinoverrucosus WP_141312464.1_Lan A1

MFVDIVRSWKDADYRLSLGSEAPAHPSGDGLTAITDEELTEVNGAGSGVLGTLGCCSCLPWYSGWTVCGLACNPGKPCKN

>Streptomyces spinoverrucosus WP_141312463.1_Lan A2

MNLVRAWKDPEYRATLSEAPANPAGLVELADDQLDGVAGGTTWACATVTLTVTVCSPTGTLCGSCQMGTRGCC

>Streptomyces filamentous Ros A1

MDIVRSWKDADYRLSLGSEAPAHPSGEGLTAITDEELTEINGAGSGVLGTLGCCSCLPWYSGWTVCGLACNPGKPCKN

>Streptomyces filamentous_Ros A2

MNLVRAWKDPEYRATLSEAPANPAGLVELADDQLDGVAGGTTWACATVTLTVTVCSPTGTLCGSCSMGTRGC**C**

> Streptomyces sp. ADI96-02 WP_124269439.1_Lan A1

MFVDIVRSWKDADYRLSLGSEAPAHPSGEGLTAITDEELTEINGAGSGVLGTLGCCSCLPWYSGWTVCGL

ACDPGKPCKN

>Streptomyces sp. ADI96-02 WP_006126649.1_Lan A2

MNLVRAWKDPEYRATLSEAPANPAGLVELADDQLDGVAGGTTWACATVTLTVTVCSPTGTLCGSCSMGTR

GCC

>Catellatospora methionotrophica WP_166378759.1_Lan A1

MNIVQAWKDPEYRASLSAEQLAALPEHPCGVVELGDDVLAHIAGARTEYLWTLGCCGGFTARETPCGSCG

ATCGGTTCGTCQTQSTCGLCTA

>Catellatospora methionotrophica WP_166378761.1_Lan A2

MDYIRAWKDPVYRASLSDEERASLPANPAGFIELSDNELDGADGGTWTPTPTITAVTGVAGCFTINGTFC

NGTCAVITVGCCG

>Micromonospora noduli WP_091399738.1_Lan A1

MNIVQAWKDPEYRASLSAEQLAALPEHPCGVVELGDDVLAHIAGARTEGLWTLGCCGGFTARETPCGSCG

ATCGGTTCGTCQTQSTCGLCTA

>Micromonospora noduli WP_091399741.1_Lan A2

MDYIRAWKDPVYRASLSPEDRASLPANPAGFVELNDQELDGADGGTWTPTPTITVVTGAAGCFSFNGTFC

NGTCAAFTAGCCG

>Micromonospora arida WP_091399738.1_Lan A1

MNIVQAWKDPEYRASLSAEQLAALPEHPCGVVELGDDVLAHIAGARTEGLWTLGCCGGFTARETPCGSCG

ATCGGTTCGTCQTQSTCGLCTA

>Micromonospora arida WP_112603060.1_Lan A2

MDYIRAWKDPVYRASLSAEDRASLPANPAGFVELNDQELDGADGGTWTPTPTITVVTGAAGCFSFNGTFC

NGTCAAFTAGCCG

**Proteobacteria**

> Archangium gephyra WP_047856213.1_LanA

MSNIDVVRAWKDEQYRMSLTTEERAQLPQNPAGMVELTDSDLEGVAGGYAEAVDIDVSVTKTSCCTYSTT

ATACC

> Vitiosangium sp. GDMCC 1.1324 WP_108076666.1_LanA

MKKEMIIRAWKDPSFRASLSDEERATIPESPSGRALTELDEGELNAIVGGKAVDLQPSTGCTGPVRATCG

IVLCSPAE

> Melittangium boletus WP_095980838.1_LanA

MSRIDIARAWKDPSYLESLSEEERALVPANPAGEIALSEDDLAVIVGGLRIQPTTVSTGTGSKNPPCQCT

CLLSD

>Cystobacter fuscus WP_095984398.1_LanA

MKKTIQAWKDEDFRLSLTEEERAQLPANPAGIVELSDEALDSLLSGGKQVSSCCWESC

> Cystobacter fuscus WP_076606333.1_LanA

MSKKVIQVWKDEDFRLSMTEEERARFPDNPAGLLELTDEALDALVHGAAGVNASCGWSSCNRTN

**Chloroflexi**

> Thermogemmatispora carboxidivorans WP_069803490.1_LanA

MKFDIVRAWKDEAYRQSLSEEELSLLPESPIGEVELTDADLEAVQGGHGNTDECNNTVAVLCLQSLAVLL

GLCNTNAAGGCL

> Thermosporothrix hazakensis WP_111318317.1_LanA

MSFDVVRAWKDEEYRNSLSAEELAMLPECPAGEMELTDADLEQVSGGCHHHANQAISFGCVNSAALACLQ

SALIGACVSAGICNVD

**Acidobacteria**

> Acidobacteria bacterium gp4 AA17 PYT03204.1_LanA

MSKADVIRAWKDPDYRGSLGASELAALPENPAGAIELTDDDLDAPEVGFATTYWTCTCTTATRQITCTF

>Acidobacteria bacterium gp4 AA12 PYT07917.1_LanA

MSHADTIRAWKDEAYRLSMSEAERERMPDHPAGLIELPDSALGEAAGGTAGSYLVCSCVGPCPTDNFGCE

TVPFAFCTSVFDFCPFFPIQIA

>Acidobacteria bacterium gp4 AA12 PYT07918.1_LanA

MCKFDVVRAWKDEDYRMSLSDAERRMLPTNPAGLIEISDSELGAVAGGEAGGISFFGVCSCFGDCMSELF

QVCSGDWMPCTIGPLFCPVFPIE

> Blastocatellia bacterium AA13 PYP89986.1_LanA

MSRLDVIRAWKDEEYRAGLSEEERSLLPENPAGTMELSSLEVTGAAANAITIFNTCDGLCTGDFAPCSLE

GGCAQSVDFGRCTMWICPTGGPFFCIPDAS

> Blastocatellia bacterium AA13 PYP90029.1_LanA

MKNHDVIRAWKDEDYRLELSQAERDLLPENPAGSIEIGGLDGLRAAGAVEGTGAMNPFEIRTCIGICTPT

QDMLCGVTVGLCSFLDPCPSSVPQF

> Blastocatellia bacterium AA13 PYP90003.1_LanA

MSHLNIVRAWKDEEYRSSLSEVDRAQLPENPAGSIDTIEGFAQEMMIAGAGSFECTCLSECVSINVACGM

TGRLDCSISFICMLPDTTPIQPFLPLFPIER

**Cyanobacteria**

> Stanieria cyanosphaera WP_015192458.1_LanA

MSNSDIIRAWKDEDYSSSLSEEQRSQLPDNPIGIVELSDEDMEIVAGGAVNINSFGACNLNSQAAIVCLS

ASGFGATC

> Stanieria cyanosphaera WP_015192459.1_LanA

MDIIRAWKDEDYYSNLNQEELRLLPENPAGIIELSDEQMEGVSGGGLQDNSFAACNINVNFNFNISVGEK

ATCNINSNNASVCISNIGGSC

>Stanieria cyanosphaera WP_015192461.1_LanA

MSNQDIVRAWKDEDYWHSLSEEMRSRLPENPAGIIELSDEQMELIVGGLKISSRGNCTINSNHSQVCITK

QNFNCLITKVIKNGVCINRVF

> Stanieria cyanosphaera WP_015192462.1_LanA

MSNQDIIRAWKDENYWNSLSEEQRSQLPENPAGITELIDIEMETIAGGKYYLMPKFPREFFEQPSTYFLT

GCDVFPNG

>Nostocales cyanobacterium HT-58-2] WP_087541463.1_LanA

MSQQDIIRAWKDEDFRNSLTEEQLSHLPENPAGILELEDEEMKNISGGYYADSKITCDVYCWYWSYFTR

>Nostocales cyanobacterium HT-58-2] WP_087541464.1_LanA

MSQQDIIRAWKDEDFRNSLTEEQLSHLPKNPAGILELEDEEMKNISGGAVSTSDIYCWYWSYFTR

>Nostoc sp. PCC 7524 WP_015136862.1_LanA

MSVDIIRAWKDEEYRQSLSTEQLQQLPANPAGLIELNDEDMSSVSGGCTTCGNPLHTPIIKCKAVFGELG

DISA

>Fischerella thermalis WP_102151618.1_LanA

MSVDIIRAWKDEEYRQSLSTEQLQQLPENPAGLIELSDEDMSSVAGGCTTCGNPLHTPIIKCKAVFGELE

ISV

>Fischerella muscicola WP_102205352.1_LanA

MSVDIIRAWKDEEYRQSLSTEQLQQLPANPAGLIELSDEDMSSVSGGCTTCGNPLHTPIIRCKPVFGELS

DISV

>Nitrolancea hollandica WP_008480453.1_LanA

MSKLDIIRAWKDEEYRLSLDETERALLPANPAGLVELTDVDLDQAAGGGFSHSNDSFSNDSFSNDSFSDS

FSGGFSHGGFSHGGFSHGGFSSSW

>Alkalinema sp. CACIAM 70d OUC14106.1_LanA

MSQENVIRFWKDESFRNSLKDRDQTELPAHPAGLIQLNDADLGSVAGGFNSCLAGSHQPVCQDRCGI

>Alkalinema sp. CACIAM 70d OUC14107.1_LanA

MSQENIIRAWKDADFRNSLSRDAKTVLPDNPAGLIQLNDTELGSIGGGDSNVPWCPIEKQLISECTCRLK

>Chamaesiphon minutus WP_015159460.1_LanA

MSHENIIRTWKDENFRNSLSKKERALLPANPAGLVELSDADLNAVAGGAKPKSTSPCCTHATK

> Chamaesiphon minutus WP_157260190.1_LanA

MSHENIIRAWKNEGFRQSLSDSNSKFRF

>Chamaesiphon minutus WP_015159456.1_LanA

MLENIKALLQNTQLQQQVKAAANLAAASELITTAGAQKGYSFTPESVAGAIGKLMLGDRELSEADLLSVA

GGAMPACCPVVRTADSKRGCGG

>Chamaesiphon minutus WP_015159458.1_LanA

MLENIKELLQNTQLQQQVKAATNLSTASELITTAAAQKGYFFTPESVAGAIGGLMLGDRELSEADLLSVA

GGAMGMCCAIGGTRSTKPD

>orf1

MSAESVGKFLERVEADEQLQEELAQVIETAASTATEGADRQGATELGQKYGFDFSSEELWAEINKRQDQVKDRQGSGELSDDELEAVAGGGEIWIATIASTIGALIGKIKW

>orf2

MSAQSVTQFLERVETDEQLQEELAQIIESAANSATDGADRQGATELGKKYGFDFSSEELWAEIKNRQDQFKERQGSGELSDEELEAVAGGGEIWITTIFTATTAIATAVIPKIKW

>orf3

MSAQSVTQFLERVETDEQLQEELAQIIESAANSATDGADRQGATELGKKYGFDFSSEELWAEIKNRQDQFKQRQGSGELSDEELEAVAGGGEIWITTVFTATAAIATAVIPKIKW

>orf4 MSTQSVTQFLKRVETDEQLQEELAQIIESAANTGADGADRQGATELGKKYGFDFSSEELWAEIKNRQDQFKQRQGSGELSDDELEAVAGGGEIWLTTVITSTVAIGKAIYPKIKW

>Synechocystis sp. PCC 7509 WP_009630785.1_LanA

MSHENIIRAWKDADFRNRLSEKERALLPKNPVGLVELTDAELGFAAGGRYNNTGDGGCSQSPHCI

> Synechocystis sp. PCC 7509 WP_009630786.1_LanA

MSHENIVRAWKDTEFCNSLSEKERLLLPENPVGLVQLTDAELGAVVGRIAAVTDKTCPALRRYC

> Synechocystis sp. PCC 7509 WP_009630787.1_LanA

MSHENIIRAWKDEEFRNSLSEKERALLPENPVGLVELTNNELGNVSGGRRNDDTLGGSYDCTYICCVTVK

RQDPL

> Synechocystis sp. PCC 7509 WP_009630790.1_LanA

MSHENIIRAWKDENYRQSLTPEEQSLLPANPAGMLELTDTQLENAAGGRKFTYGGDESCNSGIIACTLPL

ICGVEK

> Synechocystis sp. PCC 7509 WP_009630791.1_LanA

MSNENIIRAWKDREFRNSLSKQESELLPTHPAGLVELIDEDLGAAAGGIRAEDTHYMSKCIVCC

> Synechocystis sp. PCC 7509 WP_009630793.1_LanA

MNLNIKELLQNTQLQQQLKAASNLVEATKMIVTAGAEKGYFFSQESIAQVVSGLMLEEHALTESDLLAVA

GGRMPDCKCGLVYCSE

> Synechocystis sp. PCC 7509 WP_009630794.1_LanA

MILTIKKLLQNTQLQQQVKEADNLIEAIKLITNFGAKKGYSFTQDSVVQMFSRLSLEEQELSEADLLAVT

GGLQPISGGLCKTINSGTRC

> Synechocystis sp. PCC 7509 WP_009630795.1_LanA

MIQHIKHLMENNQLKQQIKESSNLVEAIKLITNAGVQQGYSFTQDSVAEIVSGLMLIEQELTEEDLLAVS

GGLRNSCTIFTSWDSIFSVLC

> Synechocystis sp. PCC 7509 WP_009630796.1_LanA

MIYNIKQLLQNTQLKQQVKESSNLVEAIKLIVNAGVQKGYTFTQENVAQVVSELILDERELSESELLAVT

GGLRGSAITGLSFYDCCW

> Chlorogloeopsis fritschii WP_016878212.1_LanA

MSNEDIIRAWKDEEYRNSLSEEHRAQLPENPAGLIELTDAEIETINGGSKRTKDSTICDLVKKTTDPTTE

CKPSSHVLTCSSDSTSLDMFSI

> Nostoc piscinale WP_083468702.1_LanA

MSNFDIIRAWKDEDYRNSLSDEQRSQLPQNPAGMVELTNTSMETVVGGNQQLATGGTVGLKKQTITVSID

VCCSTGDLPCNGKTQDLLCIVY

>Nostoc sp. MBR 210 OCQ98810.1_LanA

MSNFDIIRAWKDEDYRNSLSDEQRSQLPENPAGLIELPDAESNALSGGGCSIFTGTCGRVCERLTPQYGC

KGCDNGGLTT

>Coleofasciculus chthonoplastes WP_006100521.1_LanA

MSNLDIIRAWKDEEYRNSLSDEQRAQLPENPAGMIELSDEDMGAISGGFAASRSVSRQGGCSCSCGGNCC

SKPQIQNIAAL

>Nostoc minutum NIES26 ORF_LanA

MGQINISDIDVVRAWKDEEYRSSLTEAQRAQLPENPAGLIDIMDEETNEIIGGWSFPVITKFLRCGRVLSLTAECHCARTITGGCRCPI

>Tolypothrix sp. PCC 7910 WP_167721410.1_LanA

MSNFDIIRAWKDEDYRNSLTEEQRSQLPENPAGQIQLSDEEMEAVAGGYVGKFRLPPEFFAAPSTYFLTG

CDVFPNG

>Tolypothrix sp. PCC 7601 WP_081584166.1_LanA

MSNFDIIRAWKDEDYRNSL**S**EEQRSQLPENPAGQIQLSDEEMEAVAGGYVGKFRLPPEFFAAPSTYFLTG

CDVFPNG

>Hapalosiphon sp. MRB220 WP_053458365.1_LanA

MSQQDIIRAWKDADFRASLSQEQRSQLPKNPAGIDEIADEYLETIVGGRGCGGGTGCGTGGNCTCCGGNC

TGKL

> Nostoc carneum WP_096727142.1_LanA

MSQQDIIRAWKDREYRESLSEEQRSNLPENPAGIAELSDEVLETIAGGRGGDGGGGGTGCGTGGNCSKCN

PCSAPK

>Nostoc sp. 106C WP_086768465.1_LanA

MSQQDIIRAWKNREYRESLSEEQRSQLPENPAGIADLSDEVLETIAGGMMARDGGGGGTGCGTGGNCSRC

NPCSAPK

**B.**

**Type II roseocin homologs (Single LanM-two precursor)**

>Mobiluncus mulieris_LanA1 (WP_004018081.1/WP_103758807.1/WP_114989942.1/WP_036384001.1)

MFEFEEVDLAEVPEELLENEAGAGSAAFKTLGCCWCLPWYSSWTKCGVVCSEQYC

>Mobiluncus mulieris_ORF LanA2

MVKEPELDFVVELDDEDLDVSGGTSIPCGTLIIATLTQCFNDTLVWGSCRLGTRACC

>Actinomyces radicidentis_LanA1 WP_150118485.1 (WP_067943472.1)

MSNTPVVEVDLDELTDLELQDVAGAAGSGWMYTFGCCWCVPWYSSFTKCGLACSQGTCR

>Actinomyces radicidentis_LanA2 WP_067943476.1 (WP_067943472.1)

MHLKENPVHVVELDDDEMAVIDGGTSIPCAAFVVTTVTACWNDTALWGSCRLGTRGCC

>Actinomyces sp. Z5_LanA1 WP_111820290.1 (WP_111820291.1)

MSTTTQNAPAVIEVDLAELTDLELQSVAGAAGSGWVHTLGCCWCLPWYSSYTKCGAICKQGTCS

> Actinomyces sp. Z5_LanA2 WP_111820289.1

MSTSIPPLDIVELDDAEMSTVDGGTSLPCAGLVITTMTTCFNDTALWGSCRLGTRGCC

>Corynebacterium sp. CNJ-954_LanA1 (WP_075814817.1)

MKTTNVQVDLTEVIDEEISDVNGAASGMFGTIGCCWCVPWYSGITKCGIACGRQTC

>Corynebacterium sp. CNJ-954_LanA2 (WP_075814817.1)

MHLNHNPEKFRVPLVELDDADIDEVNGGTTSIPCGTIAIASITQCFGDSAIWGSCKLGTRACC

>Cryobacterium sp. Y57-LanA1 WP_146071102.1 (WP_104127098.1)

MNTITQWKNTSLFDVDESTHPAGRVDLTELSDFELDGINGAAGTALGGTWGCCWCVPWYSSWTQCGVICSHPGCSY

>Cryobacterium sp. Y57-LanA2 (WP_104127098.1)

MNTVIEWKNLERSGRNHPAGLVELDDVEMREVNGGTTPWCATATIIIATISACFGNTAIWGSCELGTRGCCR

**Short size roseocin homologs**

>Streptomyces CB 02056/Streptomyces xanthocidicus_LanA1 WP_049659012.1

(WP_079272258.1/WP_117485621.1)

MSDNSTARAWKDPEDAARAVAPAHPSGEVDLGLFVGGLSDEEGGSEALGTGGCCPTWATGTHC

>Streptomyces CB 02056/Streptomyces xanthocidicus_LanA2 WP_074003146.1

(WP_079272258.1/WP_117485621.1)

MSNEDIVRSWKDPETARTAPGHPVGEPDLTALSGGEEQITVYDICTTNQTGCGTCGSFSFGCC

>Streptomyces CB 02056_LanA2 (WP_079272258.1)

MSDEETVRSWKDPETARTTPGHPLGEPDLTALSGGEEPVTVFEMCTGQTACGTCGGFTFGCC

>Streptomyces CB 02056_LanA2 (WP_079272258.1)

MSNEDIVRSWKDPETARTTPGHPLGEPDLTALSGGEEQITNNDLCNNTERSWCSTCGAFTLGCC

>Streptomyces CB 02056_LanA2 (WP_079272258.1)

MSDEETVRSWKDPETARTAPGHPLGEPDLTALSGGEEAFTNNDFCSSTDQSWCGTCGSFSYGCC

>Streptomyces xanthocidicus_LanA2 WP_117485625.1 (WP_117485621.1)

MSDEETVRSWKDPETARATPGHPLGEPDLTALSGGEEPVTVFEMCTGQTACGTCGGFTFGCC

>Streptomyces xanthocidicus_LanA2 WP_117485627.1 (WP_117485621.1)

MSDEETVRSWKDPETARTTPGHPLGEPDLTALSGGEEAFTNNDFCSSTDQSWCGTCGSFSYGCC

>Streptomyces alni_LanA1_WP_093716817.1 (WP_093716811.1)

MDLDNDVVRSWKEYAPAGAPDSSPVSSIDIADVAQGGMIGGSWQWSFSCCDTTLCSDCSIN

**Another member of roseocin family, identified using *S. alni* LanM sequence (WP_093716811.1)** **as query**

>Hamadaea sp._LanA1_NUT05935.1

MNPETVRGWKDLDGDTAHPAGAIDLSAVSGGVMATNGIWTLGCCDPTANSFWICSSECTSGVC

>Hamadaea sp._LanA1_NUT05934.1 MSRDDTIRTWKNGSDLPEPQHPAGSVDLTAVIGGQQAPTSEDLFTWGCCSAKTWCGGGTCW

>Hamadaea sp._LanA1_NUT05932.1 MNQETTIAAWKGVSDQDGPANPAGELAEALTVAGGVINVISTEHVHTLGCCPGFTNEPGCWTW

>Hamadaea sp._LanA2_NUT05933.1

MSIPTSDVIRAWKDADHDDNPVGTVDLARLHGAVQAWTVDLAGCDQTIGMCTHAPCTMQCVP

>Hamadaea sp._LanA2_NUT05931.1

MSNQQLLRSWKDPEASADAVSPAGEVDLSVLTGGQQADTLLPWFCDKTIYQGTCAMGPTVGCC
